# Bioactivity Profiling and Untargeted Metabolomics of Microbiota Associated with Mesopelagic Jellyfish *Periphylla periphylla*

**DOI:** 10.3390/md21020129

**Published:** 2023-02-17

**Authors:** Ernest Oppong-Danquah, Martina Miranda, Martina Blümel, Deniz Tasdemir

**Affiliations:** 1 GEOMAR Centre for Marine Biotechnology (GEOMAR-Biotech), Research Unit Marine Natural Products Chemistry, GEOMAR Helmholtz Centre for Ocean Research Kiel, Am Kiel-Kanal 44, 24106 Kiel, Germany; 2 Faculty of Mathematics and Natural Science, Kiel University, Christian-Albrechts-Platz 4, 24118 Kiel, Germany

**Keywords:** mesopelagic zone, jellyfish, *Periphylla periphylla*, metabolomics, feature-based molecular networking, antimicrobial activity

## Abstract

The marine mesopelagic zone extends from water depths of 200 m to 1000 m and is home to a vast number and diversity of species. It is one of the least understood regions of the marine environment with untapped resources of pharmaceutical relevance. The mesopelagic jellyfish *Periphylla periphylla* is a well-known and widely distributed species in the mesopelagic zone; however, the diversity or the pharmaceutical potential of its cultivable microbiota has not been explored. In this study, we isolated microorganisms associated with the inner and outer umbrella of *P. periphylla* collected in Irminger Sea by a culture-dependent approach, and profiled their chemical composition and biological activities. Sixteen mostly gram-negative bacterial isolates were selected and subjected to an OSMAC cultivation regime approach using liquid and solid marine broth (MB) and glucose–yeast–malt (GYM) media. Their ethyl acetate (EtOAc) extracts were assessed for cytotoxicity and antimicrobial activity against fish and human pathogens. All, except one extract, displayed diverse levels of antimicrobial activities. Based on low IC_50_ values, four most bioactive gram-negative strains; *Polaribacter* sp. SU124, *Shewanella* sp. SU126, *Psychrobacter* sp. SU143 and *Psychrobacter* sp. SU137, were prioritized for an in-depth comparative and untargeted metabolomics analysis using feature-based molecular networking. Various chemical classes such as diketopiperazines, polyhydroxybutyrates (PHBs), bile acids and other lipids were putatively annotated, highlighting the biotechnological potential in *P. periphylla*-associated microbiota as well as gram-negative bacteria. This is the first study providing an insight into the cultivable bacterial community associated with the mesopelagic jellyfish *P. periphylla* and, indeed, the first to mine the metabolome and antimicrobial activities of these microorganisms.

## 1. Introduction

The marine mesopelagic zone, also known as the twilight zone, extends from water depths of 200 m to about 1000 m. It comprises about 20% of the total oceans’ volume, provides home to a highly diverse biological community including jellyfish, cephalopods, crustaceans and fish [1], and represents one of the least understood and studied ecosystems on the planet. As an example, the mesopelagic fish stock was recently calculated to be at least an order of magnitude higher than the previously estimated 10^9^ tonnes [2]. This highly diverse habitat harbors an enormous, but yet hidden potential for exploitation of high value compounds such as nutraceuticals and pharmaceuticals. Hardly any sunlight reaches the mesopelagic zone and in the open ocean, the thermocline with drastic changes in physical and chemical gradients often lies within the mesopelagic zone [3]. Additionally, the high level of predation, pressure and hypoxic conditions, shape the mesopelagic faunal community to evolve adaptation measures, which include larger eyes for good vision, skin coloration to escape predation, and establishing relationships with beneficial symbionts as well as other biochemical traits that make them promising sources of structurally and functionally unique molecules for pharmaceutical relevance [4,5].

Jellyfish are umbrella-shaped gelatinous free-swimming animals of different forms and sizes, and also inhabit different depths in the marine ecosystem [6]. They belong to the phylum Cnidaria and the subphylum Medusozoa, which is divided into four classes; Scyphozoa (true jellyfish), Cubozoa (box jellyfish), Staurozoa (stalked jellyfish) and Hydrozoa (hydroids, hydromedusae) [7]. They generally exhibit two morphological forms in their life cycle; the asexual benthic polyp phase from the larvae and the sexual planktonic medusa phase. Morphologically, jellyfish medusae are characterized by a bell-shaped umbrella, tentacles with stinging cells and oral arms [8]. They are considered important players in climate regulation as carbon sinks and nutrient cycling and are also a source of food [9,10]. They are typical consumers of zooplankton using their tentacles, oral arms and stingers to capture their prey [11]. Some jellyfish produce toxins in nematocyst organelles which are cardiotoxic, cytotoxic, neurotoxic, hemolytic and dermonecrotic and, hence, remain a public health concern with regards to recreational activities, fishery and tourism [12,13]. The outer layer of the jellyfish is sheathed in mucus mainly composed of proteins, lipids and sugars [14], making the jellyfish an attractive niche for microbial colonization. The outer body is consistently in contact with the microbial community of the surrounding sea water allowing for direct recruitment of microbial symbionts to their surfaces [15]. These epibionts confer diverse advantages to the jellyfish at all life and morphological stages [15]. As an example, settlement of the larvae of the jellyfish *Aurelia aurita* on their substrate was induced by a species of epibiotic Actinobacteria [15,16]. Downstream chemical analysis revealed that the bacterial glycolipids acylgalactosidyldiglyceride and monogalactosidyldiglyceride were responsible for larvae attachment to the substrate [15,16]. The bacterial symbionts also produce metabolites with antimicrobial and other type of activities to defend the jellyfish against pathogens and other predators [17]. The tetrodotoxin (potent neurotransmitter)-producing bacterium *Pseudoalteromonas tetraodonis* was detected being associated to tentacles of the jellyfish *Cyanea capillata* by phylogenetic analysis of the bacterial community [18].

Microorganisms associated with invertebrates have proven to be good sources of natural products for pharmaceutical applications [19,20,21]. However, only a few studies have investigated the pharmaceutical potential of jellyfish-associated microbes [22,23,24,25]. For example, the depsipeptides salinamides A-F were isolated from a *Streptomyces* sp. associated with the jellyfish *Cassiopeia xamachana*, showing significant antibacterial and anti-inflammatory activities [26]. A cyclic tetrapeptide was also isolated from the jellyfish-derived fungus *Phoma* sp. and displayed weak suppressive effect on the production of nitric oxide in murine macrophage cells [27]. These examples suggest the enormous pharmaceutical potential of jellyfish-associated microbiota.

*Periphylla periphylla* (class Scyphozoa, family Periphyllidae), also known as helmet jellyfish is widely distributed in the mesopelagic zone in several oceans [28,29]. They are able to maintain a high and sustainable population due to the lack of predators of the adult medusae [29]. Their umbrella is red in color and *P. periphylla* is capable of bioluminescence using luciferin coelenterazine [30]. *Periphylla periphylla* is one of the rare examples in Scyphozoa lacking a polyp stage, thus exhibiting a holopelagic lifestyle [28]. It is photophobic and can only be found at the ocean surface at night-time. Despite its ubiquitous distribution in the ocean, there is no comprehensive study on the microbiota associated with *P. periphylla*. Most studies have been focused on their distribution, biomass and morphology [31,32]. Not only is there paucity of research on the microbial diversity of *P. periphylla*, but also the potential of the associated microbiota for the biosynthesis of high value natural products is completely unknown.

The EU Horizon 2020 project SUMMER (Sustainable Management of Mesopelagic Resources, https://summerh2020.eu (accessed on 5 October 2022)) aims at evaluating the exploitation potential of mesopelagic resources without compromising their essential ecosystem services. One of the project objectives is the assessment of potential of cultivable microorganisms associated with mesopelagic animals for pharmaceutical applications. To this end, we report here culture-dependent microbiota associated with the umbrella of the mesopelagic jellyfish *P. periphylla* (collected during the International Ecosystem Summer Survey in the Nordic Seas (IESSNS) in the Irminger Sea, North Atlantic Ocean at a depth of 325 m). A total of 43 bacterial strains were isolated, of which 16 strains were selected based on phylogenetic differences in order to investigate their antimicrobial activities and metabolomes. In order to increase their chemical space, a ‘One-Strain-Many-Compounds (OSMAC)’ approach was employed using two culture media in two culture regimes. The initial antimicrobial and anticancer assessment of the crude extracts of these isolates allowed the selection of the most potent species for comparative UPLC–MS/MS-based untargeted metabolomics. This was performed in order to prioritize jellyfish-derived bacteria for future downstream chemical work involving purification and characterization of antimicrobial natural products. To the best of our knowledge, this is the first study reporting the culture-dependent microbial community of the mesopelagic jellyfish *P. periphylla* and also the potential of its associated microbiota for production of high value metabolites with pharmaceutical potential.

## 2. Results

### 2.1. Isolation and Characterization of Isolates

For isolation of microorganisms associated with the mesopelagic jellyfish *P. periphylla*, three different solid cultivation media, Marine agar (MA), Hastings medium (HS), and modified Wickerham medium (WSP), were used. MA is an oligotrophic medium supporting the growth of heterotrophic marine bacteria [33]. Modified Wickerham (WSP) and the nutrient rich Hastings (HS) media support the growth of both fungi and bacteria [34,35]. A total of 43 bacteria were isolated from the inner and outer surfaces of the umbrella of *P. periphylla*, but unfortunately, no fungal strain was retrieved. The isolates were identified via Sanger sequencing of the 16S rRNA gene, and based on phylogenetic distinctiveness (differences in closest relative species acc. to nucleotide BLAST), 16 bacteria belonging to 8 different genera were selected for further studies (Table 1). The complete identification of all 16 isolates incl. GenBank accession numbers are displayed in Table S1.

The selected isolates represented the phyla Pseudomonadota (formerly Proteobacteria, 12 isolates), Bacteroidota (3 isolates), and Actinomycetota (1 isolate). Hence, gram-negative prokaryotes dominate the selection and indeed, *Salinibacterium* sp. isolated from the outer umbrella surface is the only gram-positive organism. Overall, the MA medium resulted in the highest diversity of isolates as seven out of eight isolates from the outer umbrella (*Bizionia* sp., *Psychrobacter* sp., *Polaribacter* sp., *Salinibacterium* sp., *Shewanella* sp., *Pseudoalteromonas* sp. and *Psychrobacter* sp.) were retrieved from the MA medium, while only *Vibrio* sp. was isolated using HS medium. As for the inner umbrella, four isolates (*Polaribacter* sp., *Pseudoalteromonas* sp., *Vibrio* sp., *Psychrobacter* sp.) were derived from MA, two each from HS (*Aliivibrio* sp., *Pseudalteromonas* sp.) and WSP (*Psychrobacter* sp., *Shewanella* sp.), respectively.

**Figure 1:**
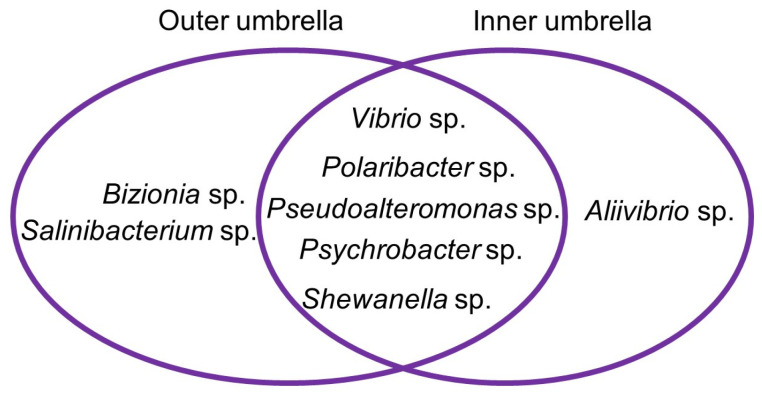
Venn diagram showing the distribution of the isolates obtained from the outer and inner umbrella of the mesopelagic jellyfish *Periphylla periphylla*.

The cultivable microbial diversity between outer and inner umbrella did not differ much. Most isolates were shared, only *Bizonia* sp. and *Salinibacterium* sp. were isolated only from the outer umbrella surface, whereas *Aliivibrio* sp. was only retrieved from the inner umbrella (Figure 1).

### 2.2. Antimicrobial and Anticancer Activity

All 16 isolates were grown in both liquid and solid culture regimes using marine broth (MB) and glucose–Yeast–malt (GYM) media, which are known to support growth and enhance metabolite production of bacteria [36]. Good growth was observed in all cultivation conditions except for *Shewanella* sp. SU126, *Polaribacter* sp. SU134, *Vibrio* sp. SU136, and *Aliivibrio* sp. SU139, which showed no growth on solid GYM.

The ethyl acetate (EtOAc) extracts of all bacterial cultures were first subjected to antimicrobial and anticancer activity assessment against eight human pathogens (*Candida albicans*, *Cryptococcus neoformans*, including the ESKAPE panel; *Enterococcus faecium*, methicillin-resistant *Staphylococcus aureus* (MRSA), *Klebsiella pneumoniae*, *Acinetobacter baumannii*, *Pseudomonas aeruginosa*, *Escherichia coli*), two fish pathogens (*Lactococcus garviae* and *Vibrio ichthyoenteri*), four cancer cell lines (human melanoma cell line A-375, colon cancer cell line HCT-116, breast cancer cell line MB-231 and the lung cancer cell line A-549), and a general toxicity against a non-cancerous cell line (human keratinocyte cell line HaCaT). None of the extracts showed toxicity towards HaCaT cells, but also no significant anticancer activity was detected at the test concentration (100 µg/mL). The crude extracts displayed varying degrees of antibacterial activity, especially against the gram-positive test strains *E. faecium*, MRSA and *L. garviae* Table S2). Isolates of the same genus, such as *Polaribacter* sp. SU134 and *Polaribacter* sp. SU124, displayed vast differences in antimicrobial activities and may represent different chemotypes. Moreover, varying antimicrobial activities were observed in extracts from different cultivation conditions (Table S2), which is indicative for different metabolomes in the different conditions. As an example, the gram-positive *Salinibacterium* sp. SU125 displayed anti-MRSA activity only when cultivated in GYM medium; and the activity in the liquid broth extract (IC_50_ value 28 µg/mL) was considerably stronger than in the solid medium extract (IC_50_ value 66.5 µg/mL). Extracts of Gammaproteobacteria *Pseudoalteromonas* sp. SU127, *Psychrobacter* sp. SU128, *Psychrobacter* sp. SU137, *Pseudoalteromonas* sp. SU140 and *Shewanella* sp. SU147 displayed the broadest activities against multiple pathogens *E. faecium*, MRSA and the fish pathogen *L. garviae* (Table S2). Only two extracts derived from *Salinibacterium* sp. SU125 in GYM-liquid and *Vibrio* sp. SU129 in MB-solid displayed activities against the fish pathogen *V. ichthyoenteri* (IC_50_ values 56.6 and 91.5 µg/mL, respectively). One Bacteroidota representative *Polaribacter* sp. SU134 showed no significant activity (IC_50_ value > 100 µg/mL) against any of the tested pathogens in all conditions (Table S2). However, the MB-liquid extract derived from another strain of the same genus, *Polaribacter* sp. SU124, showed highly potent anti-MRSA activity (IC_50_ value 7.3 µg/mL) and moderate activity against *E. faecium* (IC_50_ 67.3 µg/mL) (Table 2 and Table S2). Highly anti-MRSA active extracts were also obtained from *Shewanella* sp. SU126 on MB-solid medium and *Psychrobacter* sp. SU143 cultivated on GYM-solid medium with IC_50_ values of 8.5 and 9.9 µg/mL, respectively (Table 2). They also showed moderate activities against *E. faecium* and the fish pathogen *L. garviae* (IC_50_ values 18.7–53.4 µg/mL). The extract of *Psychrobacter* sp. SU137 grown on GYM-solid medium displayed IC_50_ values of 7.3 µg/mL against *E. faecium* and 8.1 µg/mL against MRSA (Table 2). As gram-negative bacteria are largely understudied for their potential to produce marine natural products with pharmaceutical potential, here, four highly bioactive gram-negative isolate extracts were further selected for extensive metabolomics analyses. A comparative metabolomics analysis was performed on all extracts obtained from all culture conditions (MB-solid, MB-liquid, GYM-solid and GYM-liquid) of the four selected isolates in order to gain clues on the metabolite families that may be responsible for the differential bioactivity observed in different media.

### 2.3. Metabolomics

The extracts of the selected isolates *Polaribacter* sp. SU124, *Shewanella* sp. SU126, *Psychrobacter* sp. SU143 and *Psychrobacter* sp. SU137 grown in both media (MB and GYM) and culture regimes (solid and liquid) were chemically analyzed in an untargeted metabolomics approach using feature-based molecular networking (FBMN) which allows for relative quantification [37] of metabolites. A total number of 15 extracts was analyzed as *Shewanella* sp. SU126 did not grow on GYM-solid medium. All extracts were chemically profiled by UPLC-ESI-MS/MS. Data were pre-processed with the Mzmine3 suite [38] and the output files were exported to the GNPS (global natural product social molecular networking, https://gnps.ucsd.edu (accessed on 18 November 2022)) platform and analyzed according to the FBMN workflow in both positive and negative ion modes. The ‘Merge network polarity’ workflow implemented in GNPS (https://ccms-ucsd.github.io/GNPSDocumentation/mergepolarity/ (accessed on 9 December 2022)) was used to combine networks derived from positive and negative mode analysis into a composite molecular network (MN). Each node represents consensus MS^2^ spectrum and edges between nodes represent similarity between consensus spectra. Boundaries of nodes from the negative ion mode are shown in red. Connecting edge between a positive node and a negative node is displayed in red.

**Figure 2:**
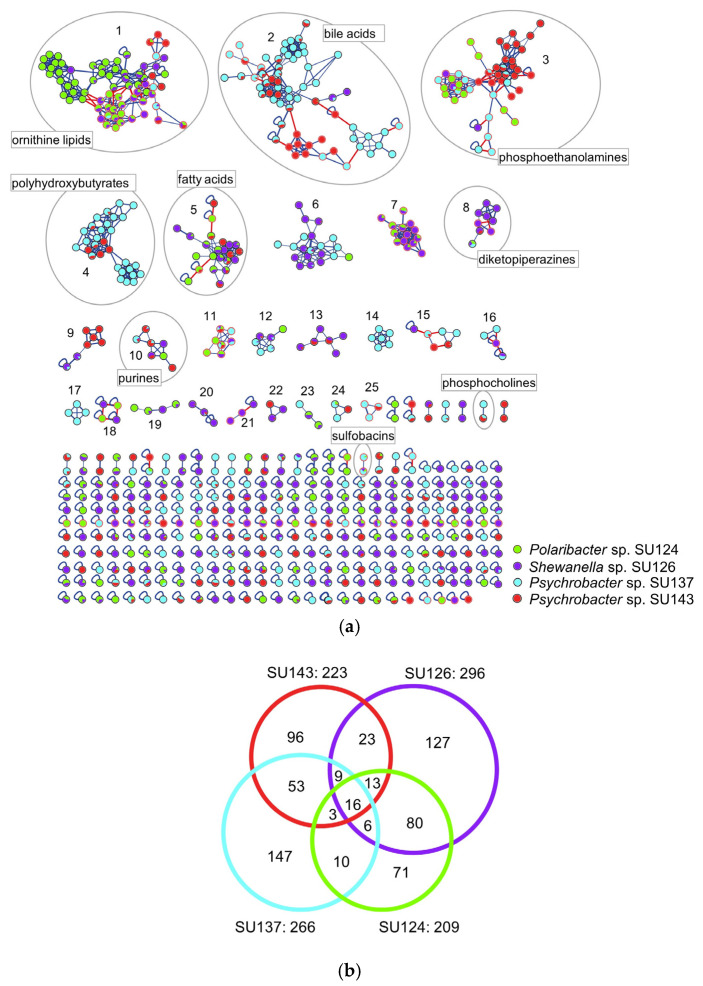
(**a**) Composite molecular network derived from positive and negative ion mode MS/MS data of the four most bioactive bacterial isolates based on solid and liquid culture extracts on MB and GYM media. Clusters (comprising nodes ≥ 3) are numbered from 1 to 25 with putative annotations. Nodes with red boundaries represent ions from negative mode, red-colored edges connect nodes originating from negative and positive ion modes. Pie charts in nodes represent relative quantities of the ions produced by the different isolates. (**b**) Euler diagram displaying the chemical diversity of the bacterial isolates; SU143-*Psychrobacter* sp., SU126-*Shewanella* sp., SU137-*Psychrobacter* sp. and SU124-*Polaribacter* sp.

The global composite MN including all extracts revealed 673 nodes organized into 25 clusters (containing three or more nodes, Figure 2a). Comparing the number of nodes produced by the four isolates, *Shewanella* sp. SU126 was the most prolific producer with 296 nodes. *Psychrobacter* sp. SU137 produced 266 nodes, followed by 233 nodes obtained from *Psychrobacter* sp. SU143 and 209 nodes from *Polaribacter* sp. SU124 (Figure 2b). Notably, *Psychrobacter* sp. SU137 displayed the most isolate-specific nodes (147), followed by *Shewanella* sp. SU126 (127 nodes) and *Psychrobacter* sp. SU143 (96 nodes). Least isolate-specific nodes were again derived from *Polaribacter* sp. SU124 (71 nodes, Figure 2b). Metabolomes of the individual isolates grown in different media are displayed in Figures S1–S4).

Overall, nine clusters were putatively annotated using the GNPS library search and other orthogonal dereplication tools such as SIRIUS/CSI:FingerID [39] and manual database searches (Figure 2a). Classes of compounds annotated include fatty acids (cluster 5), phosphoethanolamines (cluster 3), purines (cluster 10), ornithine lipids (cluster 1) and phosphocholines (two nodes), which are mostly primary metabolites [40,41,42,43,44]. They are widespread in bacteria, which explains their distribution across all four selected bacterial isolates in the clusters (Figure 2a). Only three species-specific clusters were observed; thus, clusters 14 and 17 were specific to *Psychrobacter* sp. SU137 and cluster 21 was specific to *Shewanella* sp. SU126 (Figure 2a).

Cluster 2, annotated as bile acids, was dominated by nodes from *Psychrobacter* sp. isolates SU137 and SU143. Although bile acids are uncommon products in bacteria, they are known metabolic products of the genus *Psychrobacter* [45]. The cluster was composed of nodes from both positive and negative ion modes. A negative node *m/z* 389.2695 [M − H]^−^ was annotated as 12-hydroxy-3-ketocholanic acid [45] (Figure S5). Other connecting nodes were identified as [M − H_2_O + H]^+^, [M − 2H_2_O + H]^+^ and the [2M + H]^+^ adducts. Further annotations made include dihydroxycholanic acid, dihydroxy-7-ketocholanic acid, glycodeoxycholic acid and acetylated cholic acid derivative [45], as shown in Table S3.

**Figure 3:**
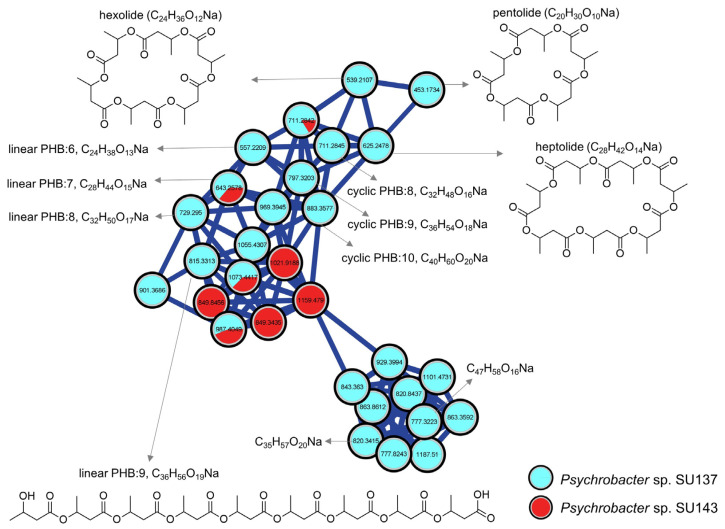
Molecular family cluster of polyhydroxybutyrates (PHB) from *Psychrobacter* sp. isolates SU137 (blue nodes) and SU143 (red nodes). Pie charts in nodes represent relative quantities of the ions produced by the different isolates.

Cluster 4 comprised 30 nodes and was putatively annotated as polyhydroxybutyrate (PHB) biopolymers [46] (Figure 3). These molecules are linear or cyclic polyesters of 3-hydroxybutyric acid (monomer) known to accumulate in some gram-negative and gram-positive bacteria [47]. Nodes in this cluster completely originated from both *Psychrobacter* spp. SU137 and SU143, and were exclusive to the positive ionization mode. Ions displayed characteristic fragment ions with a consistent difference of 86 Da corresponding to C_4_H_6_O_2_. The ion *m/z* 453.1734 [M + Na]^+^ was annotated to pentolide, the cyclic PHB from 5 units of 3-hydroxybutyric acid [46]. Based on the molecular formula prediction by MassLynx (<10 ppm), some nodes were annotated to the linear analogues as they showed extra 18 Da indicative of hydroxyl groups. Other PHBs putatively annotated include cyclic and linear members containing 6 (hexolide) [48], 7 (heptolide) [49], even 8, 9 and 10 monomer units. Many nodes in this cluster remain unannotated. However, since they are connected to nodes that are putatively annotated as oligomers, it is reasonable to assume that the unannotated nodes represent new or unreported oligomers.

Cluster 8 is comprised of nine nodes, which were produced almost solely by *Shewanella* sp. SU126, except two nodes which were also present in the other three isolates. These two nodes were annotated as the diketopiperazines cyclo-(Phe-Gly) *m/z* 205.0977 [M + H]^+^ and cyclo-(Phe-Leu) *m/z* 261.1603 [M + H]^+^ [50], and were shared by all four species.

All other clusters could not be annotated because database searches of their predicted molecular formulae returned no hits. These clusters may represent potential novel families, which will need further downstream analysis to establish their identities. The sulfonolipid sulfobacin B [51] was putatively annotated to a node originating from *Psychrobacter* sp. SU137 (62%), *Shewanella* sp. SU126 (23%) and *Polaribacter* sp. SU124 (14%). These quantitative differences are shown in the pie chart displayed in the nodes. Table S3 shows a comprehensive dereplication table of the four most bioactive isolates and their differential metabolite expression levels measured by peak areas.

**Figure 4:**
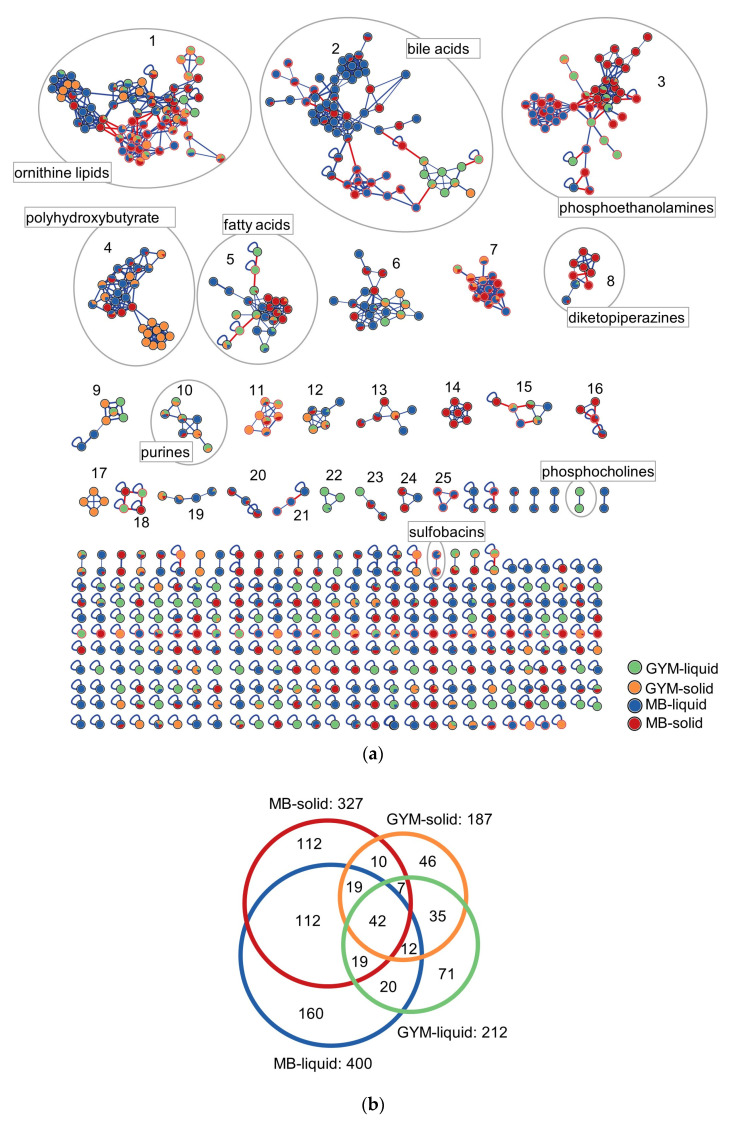
(**a**) Composite molecular network derived from positive and negative mode MS/MS data of the four most bioactive bacterial isolates displayed according to cultivation conditions; GYM-liquid, GYM-solid, MB-liquid and MB-solid media. Clusters (comprising nodes ≥ 3) are numbered from 1 to 25 with putative annotations. Nodes with red boundaries represent ions from negative mode, red-colored edges connect nodes originating from negative and positive ion modes. Pie charts in nodes represent relative quantities of the ions produced by the different isolates. (**b**) Euler diagram displaying the distribution of nodes produced by the four bacterial isolates in the different cultivation conditions.

To compare the metabolites produced in different cultivation conditions, the composite MN was displayed according to media and regime. Ions were differentially expressed in the two media and culture regimes (Figure 4). Most nodes were produced from cultivation in liquid (400 nodes) and solid (327 nodes) MB media. Considerably less nodes were detected after cultivation in GYM-liquid (212 nodes) and on GYM-solid (187 nodes, Figure 4b) media. Similarly, MB liquid extracts showed highest number of exclusive nodes (160), followed by MB-solid with 112, GYM-liquid with 71 and GYM-solid with 46 exclusive nodes (Figure 4b). These results show that cultivation of the four isolates on MB, especially in liquid regime was more prolific (ca. 79% of total nodes) than on GYM (ca. 42% of total nodes). A comparison by culture regime shows higher node numbers in liquid cultures (513, 76% of total nodes) compared to solid cultures (420 nodes, 62% of total nodes, Figure 4b). Out of the twenty-five clusters, three (14, 17 and 21, Figure 4a) were exclusively produced in specific cultivation conditions. Cluster 14 contained six nodes only produced on MB-solid by *Psychrobacter* sp. SU137 with an *m/z* range of 399.2102 to 535.3355 Da. They all displayed an intense product ion at *m/z* 165.0484 Da which could not be annotated. Cluster 17 comprised doubly charged ions only observed in MB-liquid (produced by *Psychrobacter* sp. SU137), which were annotated to a PHB class of compounds. Cluster 21, produced by *Shewanella* sp. SU126, comprised three nodes (two from negative mode and one from positive mode) and was observed only in GYM-solid. Two unannotated clusters; 7 and 11, were exclusive to the negative ion mode but from different media. Figures S1–S4 display nodes produced by the individual isolates in the different cultivation conditions.

*Polaribacter* sp. SU124 produced 106 nodes in MB-liquid, 75 in MB-solid, 88 in GYM-solid and 63 in GYM-liquid (Figure S1). Although no media/regime-specific cluster was observed, the high chemical diversity in MB-liquid aligns with the very high anti-MRSA activity recorded for this extract. Overall, 51 nodes (Figure S1) including the only diketopiperazine produced by this strain (annotated to cyclo-(Phe-Leu) were specific to MB-liquid medium and could contribute to its potent antimicrobial activity against MRSA (IC_50_ 7.3 µg/mL) compared to the MRSA activities in the other cultivation conditions (IC_50_ 20.9–23.2 µg/mL).

In *Shewanella* sp. SU126, the majority of the bioactive diketopiperazines was produced in MB-solid medium (Figure S2). Other unannotated clusters 18 and 13 were dominated by nodes derived from MB-solid. These could explain the high activity of this extract against MRSA (IC_50_ 7.3 µg/mL), as well as the moderate activities against *E. faecium* (IC_50_ 18.7 µg/mL) and the fish pathogen *L. garviae* (IC_50_ 53.4 µg/mL). Comparatively, lesser activities were observed in the other cultivation conditions (MB-liquid and GYM-liquid) with no anti- *L. garviae* activity (IC_50_ > 100 µg/mL).

The extracts of both *Psychrobacter* spp. SU137 and SU143 showed the most potent activities when grown in GYM-solid (Table 2). *Psychrobacter* sp. SU137 showed inhibitory activities against some test pathogens in all the cultivation conditions (Table 2). However, the best activities were observed against *E. faecium* (IC_50_ 7.3 µg/mL), MRSA (IC_50_ 8.1 µg/mL) and *L. garviae* (IC_50_ 20.1 µg/mL) only when cultivated in GYM-solid medium. *Psychrobacter* sp. SU137 produced the PHB cluster 17 only in GYM-solid medium as well as the majority of nodes (18 nodes) in the PHB cluster 4 (Figure S3) which may contribute to the high activities. Unlike *Psychrobacter* sp. SU137, *Psychrobacter* sp. SU143 produced few PHBs only in MB-liquid (5 nodes) and MB-solid (4 nodes) and so cannot be responsible for the potent activity observed in GYM-solid against MRSA (IC_50_ 9.9 µg/mL). The activity may, therefore, be linked to some unannotated GYM-solid specific nodes (11 nodes, Figure S4). Additionally, some singletons and clusters, such as cluster 9 and the ornithine cluster 1, showed the production of some ions (nodes) in higher titres in GYM-liquid medium than in the other media (assessed by the pie charts in the nodes, Figure S4) and may also contribute to the high activity. All annotations and their production titres (peak areas) are displayed in Table S3.

## 3. Discussion

The helmet jellyfish *P. periphylla* represents an important constituent of the zooplankton community in the mesopelagic zone. Due to their mucoidal layer covering, their surfaces represent a good carbon source, which can be readily utilized by bacteria as an energy source, hence, it hosts a good bacterial habitat. Cnidarian jellyfish can harbor bacteria relevant for aquaculture diseases, such as tenacibaculosis in fish, and were even assumed to be vectors of pathogens [52,53,54]. However, jellyfish can actively and/or passively recruit its microbial community, highlighting the dynamic and complex nature of its association with microorganisms [15]. In the present cultivation-based study, 43 isolates were retrieved from the inner and outer umbrella of *P. periphylla* using three different isolation media. As different microorganisms have different nutrient requirements, the use of diverse media for microbial isolation campaigns is always highly recommended.

Marine agar was the most suitable isolation medium used herein and is designed to support growth of mostly oligotrophic marine bacteria [55]. Similar to Marine agar, Hastings and modified Wickerham media contain yeast extract, but differ in their protein source (WSP: peptone, Hastings: tryptone), hence, were selected for this study. Furthermore, WSP contains a high amount of glucose and is therefore designed for isolation of organisms adapted to more eutrophic conditions and was originally selected for the isolation of fungi.

In line with the bacterial diversity observed in jellyfish microbial communities, our study showed a dominance of Gammaproteobacteria, representatives of Flavobacteriia and Actinomycetes [56]. The Gammaproteobacterial genera *Pseudoalteromonas* and *Vibrio* were reported to support the host defense against pathogens and fouling in other marine soft-bodied organisms [57,58]. In the current study, *Psychrobacter* sp. was the most frequently isolated Gammaproteobacterial genus associated to the umbrella of *P. periphylla*. It was previously isolated from the *Aurelia* jellyfish [56]. *Polaribacter* sp. (Bacteroidota) were previously identified in hydromedusa *Gonionemus verten* and leptomedusa *Dipleurosoma typicum* [54,59]. Other isolates in this study previously reported as part of jellyfish-associated microbiota include *Bizionia* sp. [60], *Salinibacterium* sp. [61] and *Shewanella* sp [18]. Considering that all *P. periphylla*-associated bacteria in this study were previously isolated from other jellyfish species, these associations are not considered to be species-specific. However, this study can only represent a snapshot on *P. periphylla*-associated cultivable microbial diversity as it focused on isolated microorganisms of only two *P. periphylla* individuals from one location. *Pseudoalteromonas*, *Psychrobacter* and *Shewanella* spp. have been reported in several studies as degraders of polycyclic aromatic hydrocarbons (PAHs) in crude oil [62,63]. Considering that PAH is likely to accumulate in jellyfish mucus depending on the level of anthropogenic pollution [56], it is compelling to suggest that *P. periphylla* recruits PAH-degraders for such roles as adaptation to pollution. *Aliivibrio* sp. formerly belonged to the genus *Vibrio* until its reclassification in a new genus [64]. *Aliivibrio* are known bioluminescent bacteria and are known for their symbiosis with marine organisms [65,66], e.g., with the Hawaiian bobtail squid *Euprymna scolopes* [67]. The squid uses the bioluminescence produced by the bacterium as camouflage by matching down-welling moonlight in the mesopelagic zone, a process termed as counterillumination [68,69]. The association between *Aliivibrio* sp. and the mesopelagic, bioluminescent jellyfish *P. periphylla* discovered in the current study requires further research to establish existence of a similar type of symbiosis.

No fungal isolates were recovered in this culture-based study despite use of glucose-rich WSP, usually supporting the growth of fungi [36]. Only few studies have reported jellyfish-derived fungal isolates with minimal abundances and diversity [25,27,70,71]. More so, the rather low cultivable bacterial diversity found associated with *P. periphylla* in this study is in line with previous studies on other jellyfish which employed both culture-dependent and culture-independent approaches [15,72]. It is, however, in contrast to the microbial abundance and diversity observed in fish, corals and other marine invertebrates [73,74,75,76]. It can be partly explained by the chemical defense theory; jellyfish and/or some associated microorganisms produce antimicrobial toxins that shape the microbial community to a limited number of taxa [70]. *Aurelia aurita*, as an example, produces the peptide aurelin with antimicrobial activity against both gram-negative and gram-positive bacteria [77]. Jellyfish-associated microorganisms have also produced antimicrobial compounds, such as the polyketides paecilocin A–D [25] and the neurotoxin tetrodotoxin [18], as defense mechanisms to protect jellyfish from pathogens and other microorganisms.

With the exception of *Polaribacter* sp. SU134, crude extracts of all *Periphylla* -associated bacteria showed inhibition against one or more of the tested pathogens (Table S2). This observation supports the hypothesis that these bacteria may be vital in host defense. Extracts from four isolates displayed the most potent (IC_50_ value < 10 µg/mL) inhibitory activity against at least one pathogen and were selected for in-depth comparative untargeted metabolomics analysis. All four (SU124-*Polaribacter* sp., SU126-*Shewanella* sp., SU137-/SU143- *Psychrobacter* spp.) are gram-negative bacteria, thus, supporting the enormous pharmaceutical and biotechnological potential of gram-negative bacteria, which is currently considered neglected [78].

The comparative untargeted metabolomics approach revealed diverse chemistry of the jellyfish-associated microbiota ranging from primary to secondary metabolites. The primary metabolites, ornithine lipids, purines, fatty acids and phosphoethanolamines were common to all species. Ornithine lipids (OL) are more specific to gram-negative bacteria, where they are produced in higher amounts in response to limited phosphate availability [40]. OL isolated from *Burkholderia gladioli* pv. *agaricicola* showed activity against *Bacillus megaterium* and *Escherichia coli* [79]. Diverse antimicrobial activities have further been reported for OL [80,81] and, hence, these compounds may contribute to the observed antimicrobial activities in this study. Sulfobacins are a class of sphingosine lipids first isolated from the gram-negative *Chryseobacterium* sp. without any antimicrobial activity reported [51].

In congruence with the current study, bile acid derivatives were also identified in a *Psychrobacter* sp. isolated from a sponge *Stelleta* sp. [45]. Seven bile acid derivatives were isolated, characterized by NMR and also displayed selective inhibitory activity against several human pathogens and mild antitumor activity [45]. The *Psychrobacter* spp. (SU137 and SU143) extracts obtained in this study also displayed activities against human and fish pathogens. However, they did not show antitumor activities and this could be attributed to low concentration of the bioactive bile acids in the crude extracts.

The *Psychrobacter* spp. exclusively produced the putative polyhydroxybutyrate (PHB) class of compounds [82]. Various microorganisms, such as *Pseudohalocynthiibacter* sp., *Pseudomonas* sp., *Bacillus* sp., *Burkholderia* sp. and *Cupriavidus* sp., tend to accumulate these metabolites as storage compounds [46,83]. PHB are a class of polyhydroxyalkanoates with uncommon thermal and mechanical properties rendering them good candidates for production of biodegradable plastics [47]. They have seen applications in food packaging, in biomedicine as wound dressings, orthopedic use, bone marrow scaffolds and formulation of slow release medicines among others [84,85,86]. The low molecular weight PHB pentolide, also annotated in this study, showed phytotoxic activity in a *Lemna minor* assay [46]. Synthesized and modified PHB have also shown antimicrobial activities [82,87]. The *Psychrobacter* sp. SU137 also displayed a species-specific cluster 14 which, however, remains unannotated.

The diketopiperazine cluster was largely dominated by *Shewanella* sp. SU126. An estimated 90% of gram-negative bacteria are believed to produce diketopiperazines [50]. These cyclic dipeptides have also been isolated from gram-positive bacteria, fungi and higher organisms with diverse biological activities such as antibacterial, antifungal and cytotoxic activities [88,89]. Two nodes in this cluster, annotated to cyclo-(Phe-Gly) and cyclo-(Phe-Leu), were shared by *Psychrobacter* spp. and *Polaribacter* sp. (Cluster 8, Figure 2). This cluster may also contribute to the observed activities in the extracts of these isolates.

Overall, about 68% of clusters remain unannotated, as well as many singletons. These could represent new compounds and molecular families but also show the current limitation of the natural product databases with regard to gram-negative bacteria. For example, only few compounds have been catalogued for *Psychrobacter* and *Shewanella* in DNP (dnp.chemnetbase.com), NPatlas (npatlas.org, accessed on 9 December 2022) and MarinLit (marinlit.rsc.org, accessed on 9 December 2022). No compounds from *Polaribacter* were found in these databases (access date: 1 January 2023). *Shewanella* sp. SU126 displayed the most nodes though it showed no growth in GYM-solid (Figure 2b). Based on its chemical diversity, coupled to the observed activity profile, *Shewanella* sp. SU126 will be a good candidate to be prioritized for large-scale fermentation in MB liquid medium and further isolation and characterization of its bioactive constituents.

## 4. Materials and Methods

### 4.1. Sampling and Microbial Isolation

Jellyfish *Periphylla periphylla* was collected in July 2020 in the Irminger Sea, North Atlantic Ocean, during the Icelandic part of the International Ecosystem Summer Survey in the Nordic Seas (IESSNS) with the Icelandic research vessel “Árni Friðriksson”. It was accessed by a Multpelt 832 pelagic trawl on IESSNS 2020 station 417 (net deployment position: 63°02′35″ N; 21°08′00″ W, net retrieval position: 62°57′19″ N; 21°07′63″ W) at approx. 325 m depth. Temperature at the depth of the catch was 5.8 °C. Samples were immediately processed and inoculated onboard. Sterile cotton swabs were used to swab the outer umbrella surface and afterwards the swab was placed in 500 µL sterile seawater and vortex mixed for 10 s. From the inner umbrella, a piece of surface tissue was carefully excised using sterile blades and subsequently rinsed with sterile seawater to remove contaminants. The piece was then put into 500 µL sterile seawater and homogenized by a sterile pestle, followed by vortex mixing for 10 s. For inoculation, 100 µL of the mixed samples were individually plated onto three different solid media, namely Hastings medium (HS; Na_2_HPO_4_ * 12H_2_O: 9.35 g/L, KH_2_PO_4_: 1.00 g/L, (NH_4_)_2_SO_4_: 0.5 g/L, MgSO_4_ * 7 H_2_O: 0.21 g/L, NaCl: 30 g/L, tryptone: 5 g/L, yeast extract: 3 g/L, glycerol: 2 mL/L), modified Wickerham medium (WSP; glucose monohydrate: 10 g/L, peptone from soymeal: 5 g/L, malt extract: 3 g/L, yeast extract: 3 g/L, sodium chloride: 5 g/L) and marine broth medium (MB; Difco^TM^ Marine Broth 2216: 37.4 g/L, pH 7.6 ± 0.2). Difco^TM^ Marine Broth 2216 contains peptone (5.0 g/L), yeast extract (1.0 g/L), ferric citrate (0.1 g/L), sodium chloride (19.45 g/L), magnesium chloride (5.9 g/L), magnesium sulfate (3.24 g/L), calcium chloride (1.8 g/L), potassium chloride (0.55 g/L), sodium bicarbonate (0.16 g/L), potassium bromide (0.08 g/L), strontium chloride (34.0 mg/L), boric acid (22.0 mg/L), sodium silicate (4.0 mg/L), sodium fluoride (2.4 mg/L), ammonium nitrate (1.6 mg/L) and disodium phosphate (8.0 mg/L). Inoculated plates were incubated at 6 °C to simulate the temperature in the natural habitat. Microbial growth was observed on the plates after two weeks. They were monitored daily for growth. After 21 days, morphologically distinct microbial colonies were transferred to agar plates containing fresh medium to obtain pure cultures. Pure cultures were stored in liquid nitrogen using Microbank^TM^ (PRO-LAB Diagnostics, Richmond Hill, Canada).

### 4.2. Identification of Microorganisms

All bacterial isolates were identified by the Sanger sequencing of the 16S rRNA gene. DNA was obtained either by simple freeze-and-thaw procedure as described earlier [36] or mechanical cell lysis in nuclease-free water with a Retsch mill model MM200 (Retsch GmbH, Haan, Germany). If both methods failed, the DNeasy Plant Mini Kit (Qiagen, Hilden, Germany) was used following the manufacturer’s guidelines. The 16S rRNA genes of the obtained DNA were amplified by PCR using primers Eub27f and 1387r in a previously published cycling protocol [90]. PCR products with correct fragment length were sent for Sanger sequencing at LGC Genomics (Berlin, Germany) using the primer 1387R. Resulting sequences were analysed and trimmed with ChromasPro V1.33 (Technelysium Pty Ltd., South Brisbane, Australia) and compared to the National Center for Biotechnology Information (NCBI) nucleotide database using the nucleotide BLAST function [91]. All sequences were submitted to NCBI Genbank. Isolates returning identical closest relatives after nucleotide BLAST comparison with (i) the full database and (ii) type strains were excluded from further analysis, resulting in a selection of 16 different isolates for further analysis.

### 4.3. Cultivation and Extraction

All 16 selected bacterial strains were used for cultivation and extraction. Bacterial pre-cultures were inoculated from the cryo-conservation beads and grown for seven days at 22 °C in the dark on solid and liquid MB and GYM media. Liquid pre-cultures were shaken at 120 rpm to provide equal oxygenation in the culture. Pre-cultures were then transferred to respective main cultures in liquid (shaking at 120 rpm) and solid MB and GYM media. Liquid main cultures were inoculated with an optical density (OD_600_) of 0.01 in 100 mL medium (300 mL Erlenmayer flasks). To obtain enough extract amounts, 10 replicates of liquid main cultures and 20 replicates per solid culture were cultivated. Isolates SU126, SU134, SU136 and SU139 (identified as *Shewanella* sp., *Polaribacter* sp., *Vibrio* sp. and *Aliivibrio* sp., respectively) did not grow on GYM solid medium and so were not included in further analysis. All main cultures were incubated for seven days at 22 °C in the dark before extraction. For extraction, solid cultures were homogenized in EtOAc (10 plates in 400 mL of EtOAc) with an Ultra-turrax (IKA-Werke, Staufen, Germany). The EtOAc extracts were sequentially washed with an equal volume of Milli-Q^®^ (Arium^®^, Sartorius) water in a liquid–liquid partitioning protocol to remove salts and other hydrophilic ingredients from the culture media. The pooled EtOAc extracts were evaporated to dryness, dissolved in MeOH and filtered (0.2 µm filter) into storage vials and dried under vacuum. Similarly, liquid cultures were extracted with EtOAc (500 mL of culture: 500 mL EtOAc) after homogenization by the ultra-turrax, washed with equal volume of milli Q water to obtain a clear EtOAc layer, which was dried in vacuo to obtain the crude extracts. The extracts were stored at −20 °C prior to further analysis.

### 4.4. LC–MS/MS Sample Analysis

Crude extracts (1 mg/mL) of the selected isolates were analysed by ultra-high-performance liquid chromatography (UHPLC, Acquity UPLC I-Class System, Waters) coupled to a quadrupole-time-of-flight mass spectrometer (Xevo G2-XS QToF, Waters) operated in fast data-dependent acquisition mode (LC–MS/MS in fast DDA mode) controlled by MassLynx version 4.2. Chromatographic separation was achieved on an Acquity HSS T3 C18 column (High Strength Silica C18, 1.8 mm, 100 × 2.1 mm, Waters) at a temperature of 40 °C. The mobile phase was composed of a mixture of (A) water with 0.1% formic acid (*v*/*v*) and (B) acetonitrile with 0.1% formic acid and pumped at a rate of 0.4 mL/min at the following gradient: 0.00–11.5 min, gradient from 1% to 99% B; 11.50–14.50 min, isocratic 99% B; back to initial condition in 0.1 min and followed by reconditioning phase until minute 16. Mass spectrometry data, *m/z* 50–1200 Da, was acquired with an electrospray ionization (ESI) source in both positive and negative ion modes with the following parameters: capillary voltage, 3000 V; source temperature 150 °C; desolvation temperature 500 with sampling cone and source offset at 40 and 80, respectively. The MS/MS experiments were carried out in tandem with up to five MS^2^ scans of the most abundant ions per MS^1^ scan in a data-dependent mode. Collison energy was ramped from 6 to 60 eV (Low CE) and from 9 to 80 eV (high CE). As controls, extracts from non-inoculated culture media (MB and GYM) and solvent (MeOH) were run under the same conditions.

### 4.5. Molecular Networking and Spectral Library Search

The UPLC-MS^2^ raw data were converted to mzxml format and processed with MZmine3 [38,92]. The output files (.csv and mgf) were exported to GNPS (https://gnps.ucsd.edu (accessed on 18 November 2022) [93]) and networks created with the feature-based molecular networking (FBMN) workflow [37]. The workflow assumed parameters including a precursor ion mass tolerance 0.05 Da, MS/MS fragment ion tolerance 0.05 Da, minimum pairs cosine 0.7 and minimum matched fragment ions 5, to generate the MN. The spectra in the MN were searched against GNPS spectral libraries [93,94] and only hits with scores above 0.7 and at least 6 matched peaks were kept. The resulting MNs were visualized using Cytoscape version 3.8.2 [95], and displayed with ‘directed’ style, with the edges modulated by cosine score. To simplify analysis of the network, only nodes representing ions observed between retention times of 0.3–14.30 min were considered. Nodes originating from the cultivation media (MB and GYM) and solvents (MeOH) were removed from the MN. Node colors were mapped based on the source of the spectra files. Further in silico annotations were done by Sirius/CSI:Finger-ID [39]. Only annotations confirmed by manual dereplication are displayed. Manual dereplication involved predicting the molecular formula of parent ions using MassLynx version 4.2 and then searching them against databases, such as the Dictionary of Natural Product (DNP, https://dnp.chemnetbase.com, accessed on 12 December 2022), MarinLit (https://marinlit.rsc.org/, accessed on 12 December 2022), SciFinder (https://scifinder-n.cas.org, accessed on 12 December 2022), NPAtlas (https://www.npatlas.org/, accessed on 12 December 2022) and COCONUT (https://coconut.naturalproducts.net/, accessed on 12 December 2022). Hits were further affirmed by their source and comparison of the experimental product ions to in silico fragments generated from the CFM-ID web server [96], if they lacked MS^2^ spectra reference data. Annotations were further assigned confidence levels from 1 to 4 based on the four levels of metabolite identification [97].

### 4.6. Antimicrobial and Anticancer Activity

All crude extracts were assessed for inhibitory activities against human and economically relevant fish pathogens as well as several cancer cell lines. Human pathogens included the ESKAPE panel (*Enterococcus faecium*, Efm, DSM 20477; methicillin-resistant *Staphylococcus aureus*, MRSA, DSM 18827; *Klebsiella pneumoniae*, Kp, DSM 30104, *Acinetobacter baumannii*, Ab, DSM 30007; *Pseudomonas aeruginosa*, Ps, DSM 1128 and *Escherichia coli*, Ec, DSM 1576), and the fish pathogenic bacteria *Lactococcus garviae* (Lg, DSM 20684) and *Vibrio ichthyoenteri* (Vi, DSM 14397). Cancer cell lines included the human melanoma cell line A-375, colon cancer cell line HCT-116, breast cancer cell line MB-231 and the lung cancer cell line A-549. To assess general toxicity, the human non-cancer keratinocyte cell line HaCaT was included. Test pathogens and cell lines were purchased from Leibniz Institute DSMZ-German Collection of Microorganisms and Cell Cultures (Braunschweig, Germany) and CLS Cell Lines Service (Eppelheim, Germany). Bioactivity testing was conducted at an effective test concentration of 100 µg/mL from crude extracts prepared at a concentration of 20 mg/mL in dimethyl sulfoxide (Carl Roth). The assays were performed by the broth dilution approach in 96-well microtiter plates, as previously described [98,99]. The cytostatic drug doxorubicin was used as positive control for the cancer cell lines. Other positive controls were amphotericin (Cn), ampicillin (Efm, Lg), chloramphenicol (MRSA, Ec, Kp, Vi), doxycycline (Ab), nystatin (Ca) and polymyxin B (Psa). Cultivation media and 0.5% DMSO were tested as negative controls. IC_50_ values were further calculated by Excel, for extracts that showed percentage inhibition of >50% at the initial test concentration.

## 5. Conclusions

The cultivable bacterial community associated with the mesopelagic jellyfish *P. periphylla* was found to be dominated by gram-negative Gammaproteobacteria. Extracts of these microorganisms showed antimicrobial activities against human and fish pathogens and displayed diverse chemistry including lipids, diketopiperazines and PHBs assessed by untargeted metabolomics approach. Our results suggest a high potential of *P. periphylla*-associated microbiota for the discovery of natural products for biomedical applications. Among the four most bioactive strains, *Shewanella* sp. SU126 was the most chemically rich and it will be prioritized for large scale fermentation followed by natural product isolation in future. This is the first study providing an insight into the cultivable bacterial community associated with the mesopelagic jellyfish *P. periphylla* and also the first to mine the metabolome and antimicrobial activities of these microorganisms.

## Figures and Tables

**Table 1:** Identity and taxonomic classification of 16 selected bacterial isolates obtained from the umbrella surfaces of the jellyfish *P. periphylla*. Taxonomic assignment followed the nomenclature of the List of Prokaryotic Names Standing in Nomenclature (LPSN, https://lpsn.dsmz.de (accessed on 15 December 2022)) at the German Collection of Microorganisms and Cell Cultures (DSMZ, Braunschweig Germany). MA: Marine Agar, HS: Hastings medium, WSP: Wickerham medium.

Source	Isolate Number	Isolation Medium	Genus	Class	Phylum
Outer umbrella	SU122	MA	*Bizionia* sp.	Flavobacteriia	Bacteroidota
	SU123	MA	*Psychrobacter* sp.	Gammaproteobacteria	Pseudomonadota
	SU124	MA	*Polaribacter* sp.	Flavobacteriia	Bacteroidota
	SU125	MA	*Salinibacterium* sp.	Actinomycetes	Actinomycetota
	SU126	MA	*Shewanella* sp.	Gammaproteobacteria	Pseudomonadota
	SU127	MA	*Pseudoalteromonas* sp.	Gammaproteobacteria	Pseudomonadota
	SU128	MA	*Psychrobacter* sp.	Gammaproteobacteria	Pseudomonadota
	SU129	HS	*Vibrio* sp.	Gammaproteobacteria	Pseudomonadota
Inner umbrella	SU134	MA	*Polaribacter* sp.	Flavobacteriia	Bacteroidota
	SU135	MA	*Pseudoalteromonas* sp.	Gammaproteobacteria	Pseudomonadota
	SU136	MA	*Vibrio* sp.	Gammaproteobacteria	Pseudomonadota
	SU137	MA	*Psychrobacter* sp.	Gammaproteobacteria	Pseudomonadota
	SU139	HS	*Aliivibrio* sp.	Gammaproteobacteria	Pseudomonadota
	SU140	HS	*Pseudoalteromonas* sp.	Gammaproteobacteria	Pseudomonadota
	SU143	WSP	*Psychrobacter* sp.	Gammaproteobacteria	Pseudomonadota
	SU147	WSP	*Shewanella* sp.	Gammaproteobacteria	Pseudomonadota

**Table 2:** The IC_50_ values (in µg/mL) of the four selected strain extracts against ESKAPE pathogens Efm: *Enterococcus faecium*; MRSA: methicillin-resistant *Staphylococcus aureus* and the fish pathogen Lg: *Lactococcus garviae*. Positive controls: ampicillin for Efm and Lg, chloramphenicol for MRSA. n.t: not tested because of the lack of extract. Most potent bioactivities are boldened.

Isolate	Medium-Regime	Efm	MRSA	Lg
***Polaribacter* sp. SU124**	MB-liquid	67.3	**7.3**	>100
	MB-solid	>100	20.9	>100
	GYM-liquid	>100	80.8	>100
	GYM-solid	>100	23.2	>100
***Shewanella* sp. SU126**	MB-liquid	59.9	20.7	>100
	MB-solid	18.7	**8.5**	53.4
	GYM-liquid	>100	>100	>100
	GYM-solid	n.t	n.t	n.t
***Psychrobacter* sp. SU137**	MB-liquid	37.8	21.3	69.6
	MB-solid	19.4	18.7	43.3
	GYM-liquid	18.7	18.5	56.2
	GYM-solid	**7.3**	**8.1**	20.1
***Psychrobacter* sp. SU143**	MB-liquid	>100	20.9	>100
	MB-solid	>100	25.7	>100
	GYM-liquid	20.5	20.2	>100
	GYM-solid	39.6	**9.9**	>100
**Positive control**		1.6	3.1	0.5

## Data Availability

The MS data used for the molecular networking analysis were deposited in the MassIVE Public GNPS database under the accession number MSV000091067. The molecular networking job can be publicly accessed at https://gnps.ucsd.edu/ProteoSAFe/status.jsp?task=ee891880347d44b994a0b1d7c7646621 (accessed on 20 January 2023) 16S rRNA gene sequences of selected isolates are available at Genbank and were assigned accession numbers: OQ259915–OQ259930.

## Supplementary Materials

The following supporting information can be downloaded at: https://www.mdpi.com/article/10.3390/md21020129/s1, Figures S1–S4: Molecular networks highlighting nodes produced in the different cultivation conditions by the four most bioactive bacteria; *Polaribacter* sp. SU124, *Shewanella* sp. SU126, *Psychrobacter* sp. SU137 and *Psychrobacter* sp. SU143; Figure S5: CFM-ID predicted MS/MS spectrum annotation of 12-hydroxy-3-ketocholanic acid; Table S1: Identification of the 16 *Periphylla*-associated bacteria; Table S2: Bioactivity (IC_50_ values in µg/mL) of the 16 bacterial strain extracts against ESKAPE pathogens Efm: *Enterococcus faecium*; MRSA, methicillin-resistant *Staphylococcus aureus* and the fish pathogens Lg: *Lactococcus garviae* and Vi: *Vibrio ichthyoenteri;* Table S3: Putative annotations of metabolites produced by the selected four bioactive isolates.
