# Supplementary material for: Bioactivity Profiling and Untargeted Metabolomics of Microbiota Associated with Mesopelagic Jellyfish Periphylla periphylla
Source: Mar Drugs. 2023 Feb 17;21(2):129. doi: 10.3390/md21020129 (PMC9958851; doi:10.3390/md21020129)
Supplement: Supplementary file 1 [file marinedrugs-21-00129-s001.zip › marinedrugs-2203539-supplementary.pdf]

Supporting Information

Bioactivity profiling and untargeted metabolomics of microbiota associated with mesopelagic jellyfish *Periphylla periphylla*

Ernest Oppong-Danquah [1,†], Martina Miranda[1,†], Martina Blümel [1] and Deniz Tasdemir [1,2*]

[1] GEOMAR Centre for Marine Biotechnology (GEOMAR-Biotech), Research Unit Marine Natural Products Chemistry, GEOMAR Helmholtz Centre for Ocean Research Kiel, Am Kiel-Kanal 44, 24106 Kiel, Germany; eoppong-danquah@geomar.de; martinamiranda0608@gmail.com; mbluemel@geomar.de
[2] Faculty of Mathematics and Natural Science, Kiel University, Christian-Albrechts-Platz 4, 24118 Kiel, Germany
* Correspondence: dtasdemir@geomar.de; Tel.: +49-431-6004430; ORCID ID: 0000-0002-7841-6271
† These authors have contributed equally

List of Figures Page

Figure S1. Global molecular network highlighting nodes (ions) produced in the different cultivation conditions (GYM-liquid, GYM-solid, MB-liquid and MB-solid) by *Polaribacter* sp. and Venn diagram displaying the distribution of ions according to different cultivation conditions. 3

Figure S2. Global molecular network highlighting nodes (ions) produced in the different cultivation conditions (GYM-liquid, GYM-solid, MB-liquid and MB-solid) by *Shewanella* sp. SU126 and Venn diagram displaying the distribution of ions according to different cultivation conditions. 4

Figure S3. Global molecular network highlighting nodes (ions) produced in the different cultivation conditions (GYM-liquid, GYM-solid, MB-liquid and MB-solid) by *Psychrobacter* sp. SU137 and Venn diagram displaying the distribution of ions according to different cultivation conditions. 5

Figure S4. Global molecular network highlighting nodes (ions) produced in the different cultivation conditions (GYM-liquid, GYM-solid, MB-liquid and MB-solid) by *Psychrobacter* sp. SU143 and Venn diagram displaying the distribution of ions according to different cultivation conditions. 6

Figure S5. CFM-ID predicted MS/MS spectrum annotation of 12-hydroxy-3-ketocholanic acid (*m/z* $[M-H]^-$ 389.2691) acquired by UPLC–QToF–MS/MS in negative ion mode. 7

List of Tables

Table S1. Identification of 16 selected bacteria associated to the umbrella of P. *periphylla* according to the BLAST results from the NCBI nucleotide database 8

Table S2. The IC_{50} values (in µg/mL) of the 16 bacterial strain extracts against ESKAPE pathogens Efm: *Enterococcus faecium*; MRSA, methicillin-resistant *Staphylococcus aureus* and the fish pathogens Lg: *Lactococcus garviae* and Vi: *Vibrio ichthyoenteri*. 9

Table S3. Putative annotation of metabolites produced by *Psychrobacter* sp. SU143, *Psychrobacter* sp. SU137, *Shewanella* sp. SU126 and *Polaribacter* sp. SU124 in the liquid and solid regime of MB and GYM media. 11

References 20

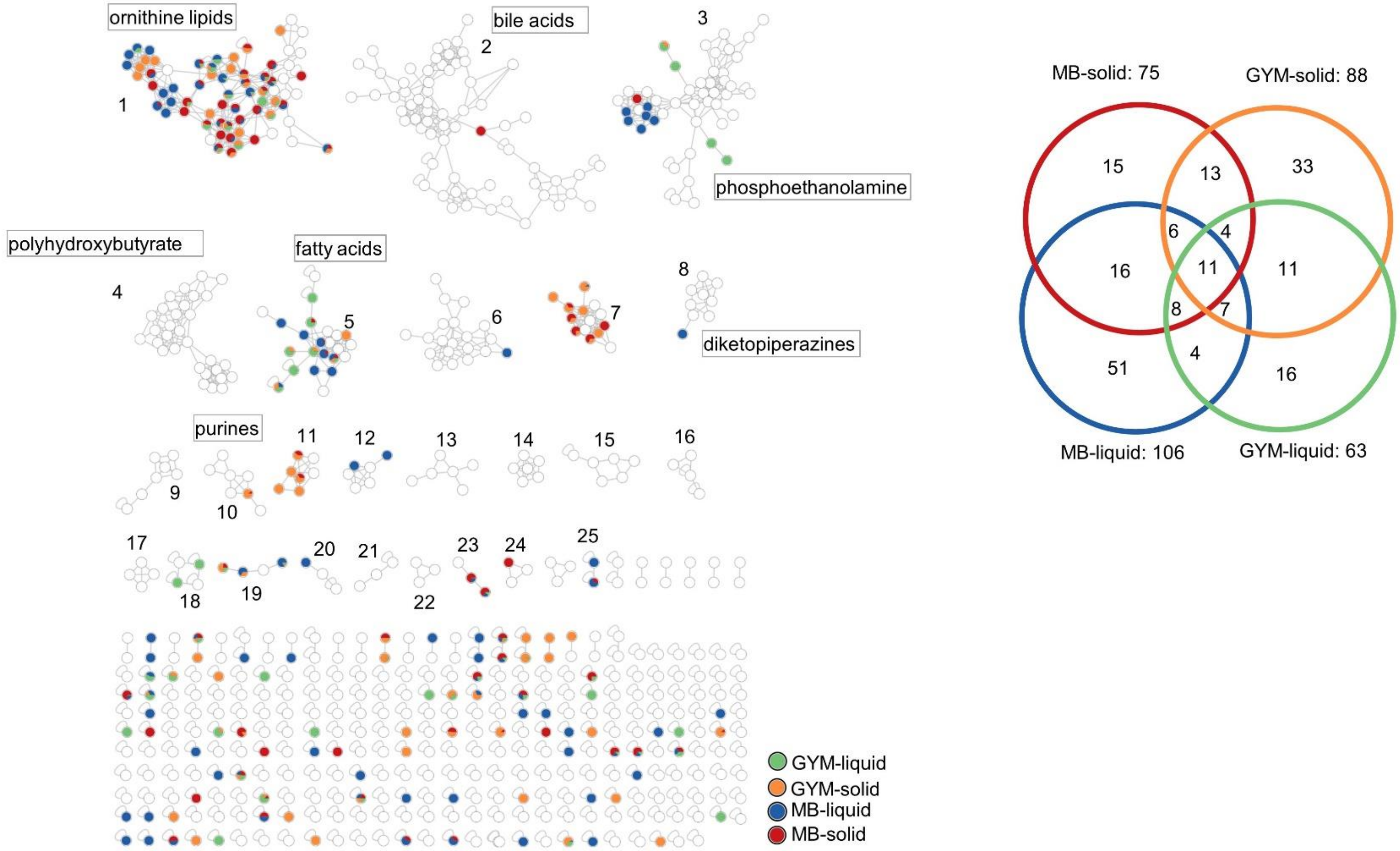

(a)

(b)

Figure S1. (a) Global molecular network highlighting nodes (ions) produced in the different cultivation conditions (GYM-liquid, GYM-solid, MB-liquid and MB-solid) by *Polaribacter* sp. Clusters (comprising nodes ≥ 3) are numbered 1 to 25 with putative annotations if any. Image is a merged MN from both positive and negative polarities. Non-colored nodes originate from the other isolates *Shewanella* sp. SU126, *Psychrobacter* sp. SU137 and *Psychrobacter* sp. SU143. (b) Venn diagram displaying the distribution of ions according to different cultivation conditions.

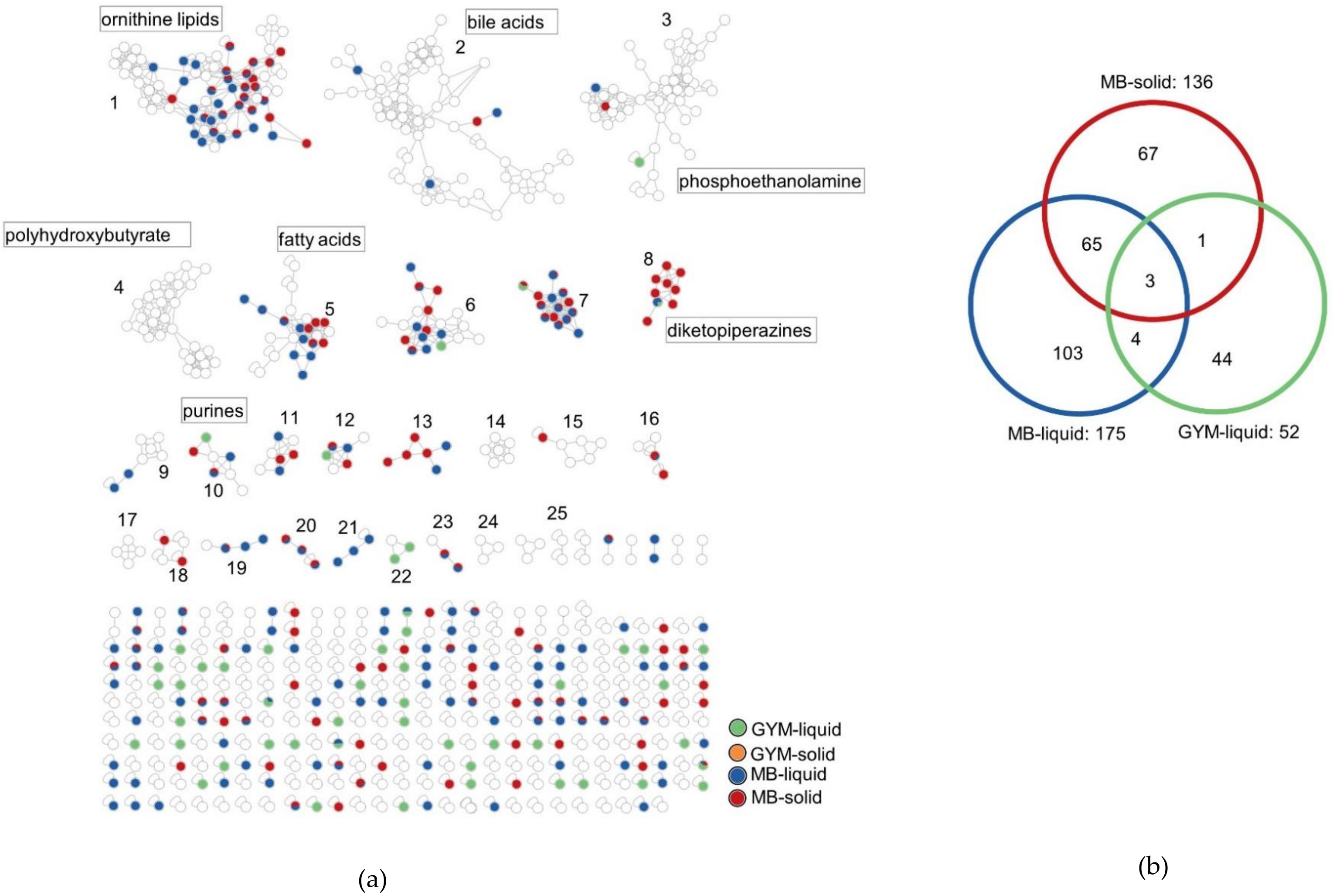

(a) (b)

Figure S2. (a) Global molecular network highlighting nodes (ions) produced in the different cultivation conditions (GYM-liquid, GYM-solid, MB-liquid and MB-solid) by *Shewanella* sp. SU126. Clusters (comprising nodes ≥ 3) are numbered 1 to 25 with putative annotations if any. Image is a merged MN from both positive and negative polarities. Non-colored nodes originate from the other isolates *Polaribacter* sp. SU124, *Psychrobacter* sp. SU137 and *Psychrobacter* sp. SU143. (b) Venn diagram displaying the distribution of ions according to different cultivation conditions.

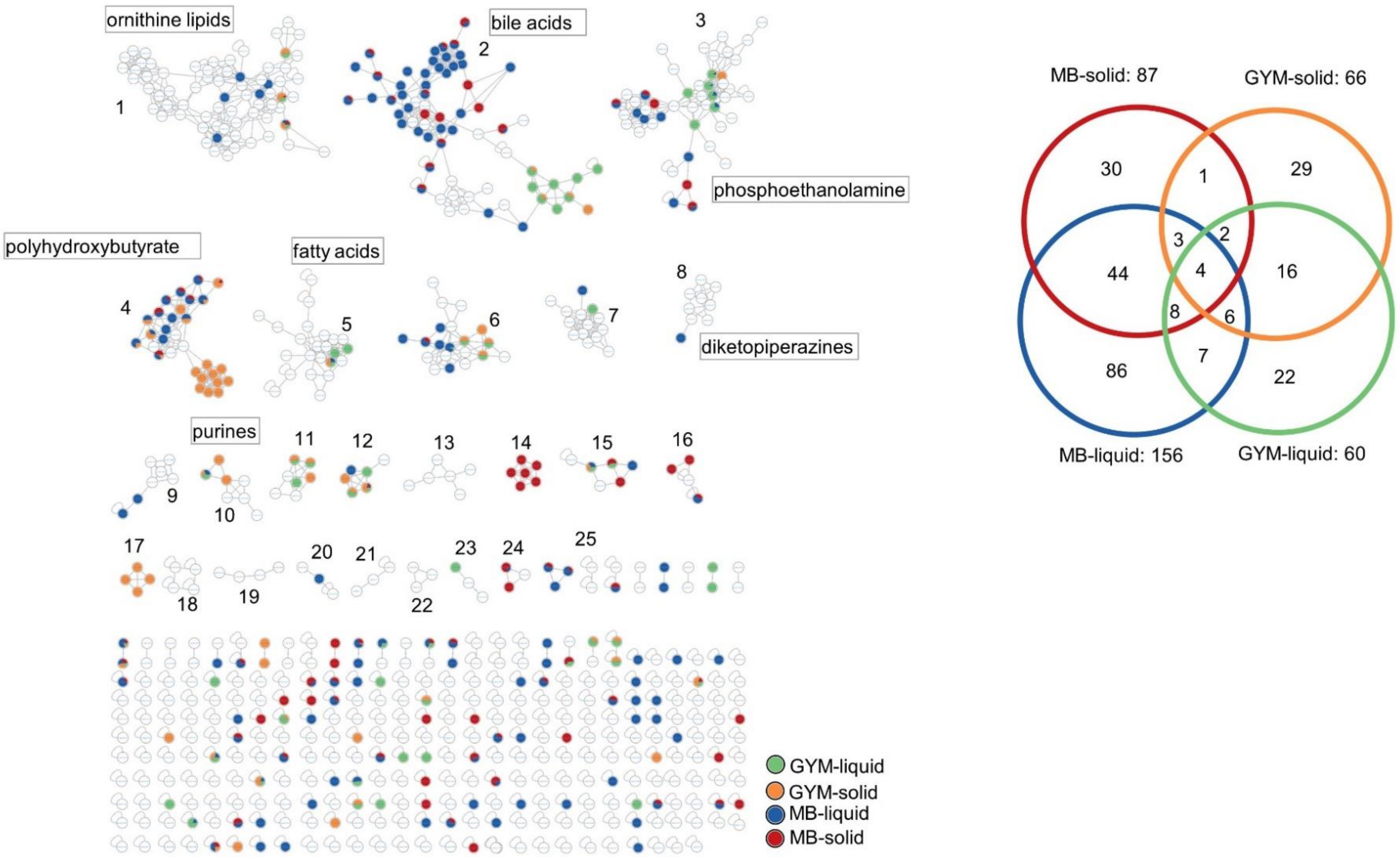

(a) (b)

Figure S3. (a) Global molecular network highlighting nodes (ions) produced in the different cultivation conditions (GYM-liquid, GYM-solid, MB-liquid and MB-solid) by *Psychrobacter* sp. SU137. Clusters (comprising nodes ≥ 3) are numbered 1 to 25 with putative annotations if any. Image is a merged MN from both positive and negative polarities. Non-colored nodes originate from the other isolates *Polaribacter* sp. SU124, *Shewanella* sp. SU126, and *Psychrobacter* sp. SU143. (b) Venn diagram displaying the distribution of ions according to different cultivation conditions.

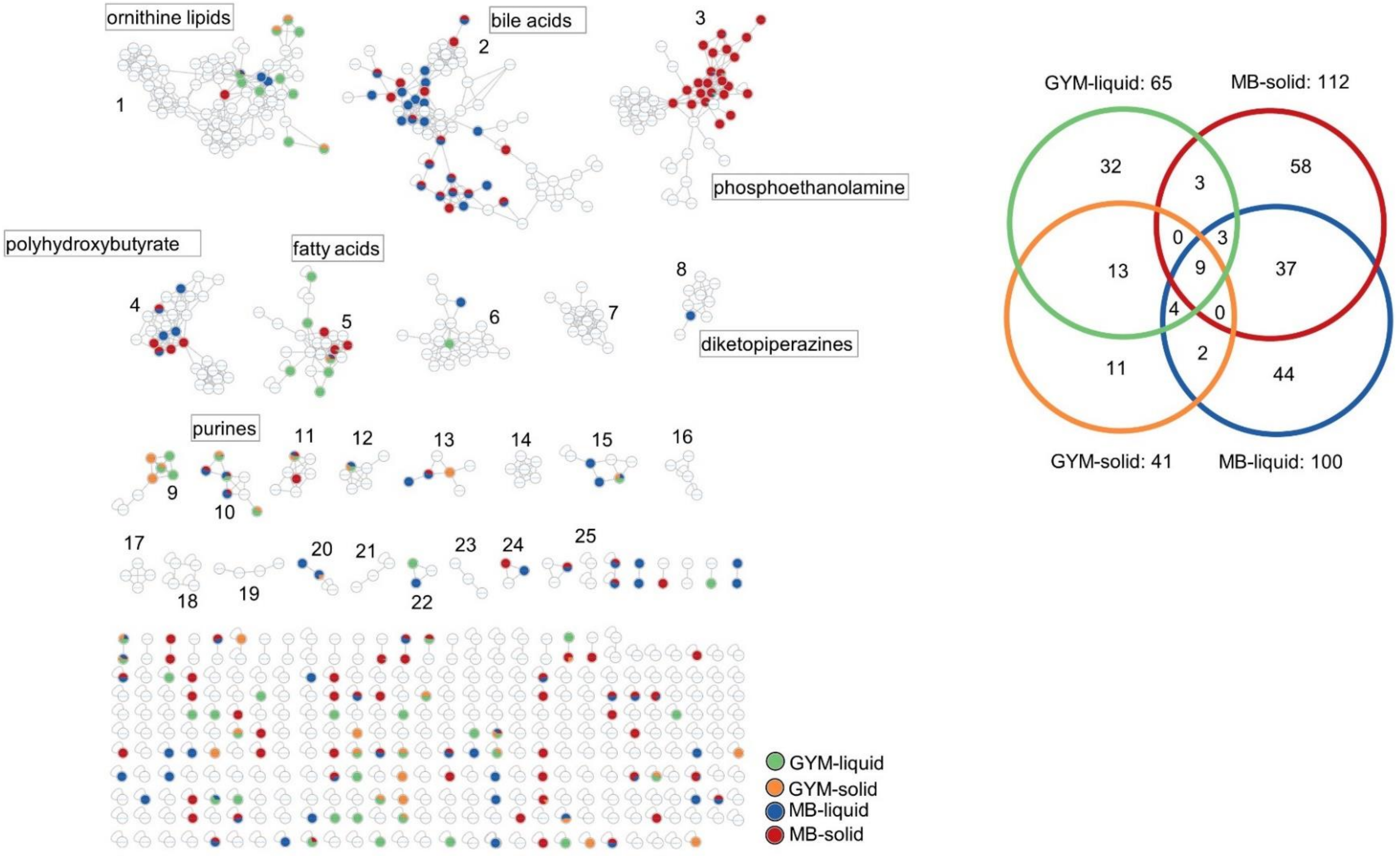

(a) (b)

Figure S4. (a) Global molecular network highlighting nodes (ions) produced in the different cultivation conditions (GYM-liquid, GYM-solid, MB-liquid and MB-solid) by *Psychrobacter* sp. SU143. Clusters (comprising nodes ≥ 3) are numbered 1 to 25 with putative annotations if any. Image is a merged MN from both positive and negative polarities. Non-colored nodes originate from the other isolates *Polaribacter* sp. SU124, *Shewanella* sp. SU126, and *Psychrobacter* sp. SU137. (b) Venn diagram displaying the distribution of ions according to different cultivation conditions.

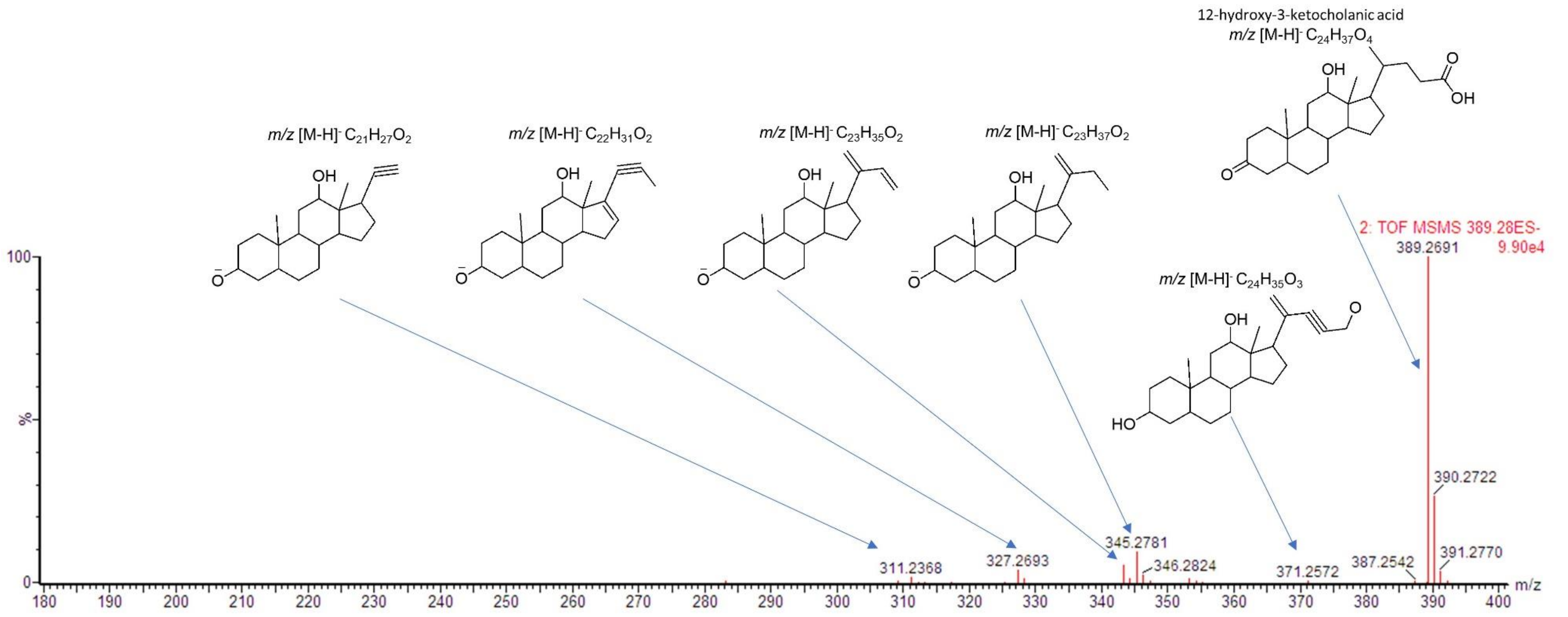

Figure S5. CFM-ID predicted MS/MS spectrum annotation of 12-hydroxy-3-ketocholanic acid (*m*/*z* [M-H]$^-$ 389.2691) acquired by UPLC–QToF–MS/MS in negative ion mode. 7

Table S1. Identification of 16 selected bacteria associated to the umbrella of P. *periphylla* according to the BLAST results from the NCBI nucleotide database. The table displays the first 3 hits from two BLAST searches, one against all database entries and the second against type strains only. MB: Marine broth; HS: Hastings medium, WSP: Wickerham medium, %: percent similarity to database hit, Acc. No.: accession numbers

Strain ID	Source	Isolation Medium	Seq. length	BLAST result (highly similar)	%	Acc. No.	BLAST result TYPE strain (highly similar)	%	Acc. No.	Acc. No. isolates
SU122	Outer umbrella	MB	764	*Bizionia* sp. strain SER20<br>*Bizionia berychis* strain RA3-3-1<br>*Bizionia* sp. BS-22B	100<br>100<br>99.9	MK660308.1<br>NR_163629.1<br>KX000020.1	*Bizionia berychis* strain RA3-3-1<br>*Bizionia fulviae* strain EM7<br>*Bizionia paragorgiae* strain KMM 6029	100<br>98.4<br>96.9	NR_163629.1<br>NR_137258.1<br>NR_025827.1	OQ259915
SU123	Outer umbrella	MB	728	*Psychrobacter* sp. BM06<br>*Psychrobacter* sp. strain SXS3-2<br>*Psychrobacter glacincola* CsDWF-377	99.6<br>99.2<br>99.0	KP172202.1<br>MG383483.1<br>MT415118.1	*Psychrobacter arcticus* strain 273-4<br>*Psychrobacter glacincola* DSM 12194<br>*Psychrobacter cryohalolentis* K5	99.0<br>99.0<br>98.8	NR_075054.1<br>NR_042076.1<br>NR_075055.1	OQ259916
SU124	Outer umbrella	MB	913	*Polaribacter atrinae* strain BM-5<br>*Polaribacter atrinae* strain WP25<br>*Polaribacter* sp. KJF13-4	99.9<br>99.9<br>99.1	MH550132.1<br>NR_133820.1<br>JQ800223.1	*Polaribacter atrinae* strain WP25<br>*Polaribacter sejongensis* KOPRI 21160<br>*Polaribacter staleyi* strain 10Alg 139	99.9<br>98.8<br>98.0	NR_133820.1<br>NR_109324.1<br>NR_159336.1	OQ259917
SU125	Outer umbrella	MB	849	*Leifsonia* sp. W3<br>*Leifsonia* sp. C6<br>*Salinibacterium* sp. strain 1664	100<br>100<br>99.9	FJ424506.1<br>FJ539111.1<br>FJ539111.1	*Salinibacterium amurskyense* KMM 3673<br>*Salinibacterium amurskyense* KCTC 9931<br>Leifsonia *rubra* strain CMS 76R	99.4<br>99.4<br>97.6	NR_041932.1<br>MT759917.1<br>MW228823.1	OQ259918
SU126	Outer umbrella	MB	770	*Shewanella* sp. HM13<br>*Shewanella* sp. strain Arc7-138<br>*Shewanella livingstonensis* LMG 19866	100<br>100<br>100	LC461000.1<br>MN784327.1<br>MK131328.1	*Shewanella livingstonensis* strain LMG 19866<br>*Shewanella vesiculosa* strain M7<br>*Shewanella livingstonensis* strain NF22	100<br>100<br>99.9	CP034015.1<br>NR_042710.1<br>NR_025443.1	OQ259919
SU127	Outer umbrella	MB	996	*Pseudoalteromonas* sp. strain L13<br>*Pseudoalteromonas* sp. strain H34<br>*Pseudoalteromonas undina* strain W14	100<br>100<br>100	MN889155.1<br>MN889141.1<br>MN746144.1	*Pseudoalteromonas tetraodonis* strain GFC<br>*Pseudoalteromonas issachenkonii* KCTC 12958<br>*Pseudoalteromonas spiralis* strain Te-2-2	100<br>99.9<br>99.8	CP011041.1<br>CP013350.1<br>NR_114801.1	OQ259920
SU128	Outer umbrella	MB	704	*Psychrobacter* sp. strain UP<br>*Psychrobacter fozii* strain D4037<br>*Psychrobacter* sp. KJF3-37	100<br>100<br>99.9	MN420817.1<br>FJ161263.1<br>JQ800055.1	*Psychrobacter fjordensis* strain BSw21516B<br>*Psychrobacter fozii* strain NF23<br>*Psychrobacter aquimaris* strain SW-210	99.3<br>99.3<br>99.0	NR_148330.1<br>NR_025531.1<br>NR_113805.1	OQ259921
SU129	Outer umbrella	HS	908	*Vibrio* sp. strain Arc7-210<br>*Vibrio alginolyticus* strain K08M4<br>*Vibrio atlanticus* strain LGP32	100<br>100<br>100	MN784332.1<br>CP017916.1<br>FM954972.2	*Vibrio kanaloae* strain LMG 20539<br>*Vibrio lentus* strain CIP 107166<br>*Vibrio atlanticus* strain VB 11.11	99.9<br>99.9<br>99.7	NR_042468.1<br>NR_114982.1<br>NR_116067.1	OQ259922
SU134	Inner umbrella	MB	985	*Polaribacter* sp. NF3-11<br>*Polaribacter* sp. BSw21683<br>*Polaribacter glomeratus* strain KOPRI_22229	98.5<br>98.4<br>98.4	FJ196052.1<br>JQ069958.1<br>EU000227.1	*Polaribacter vadi* strain LPB0003<br>*Polaribacter undariae* strain W-BA7<br>*Polaribacter sejongensis* strain KOPRI 21160	98.0<br>98.0<br>98.0	NR_156039.1<br>KM458974.1<br>NR_109324.1	OQ259923
SU135	Inner umbrella	MB	765	*Pseudoalteromonas neustonica* strain CeD-1<br>*Pseudoalteromonas neustonica* SJOD-M-26<br>*Pseudoalteromonas* sp. strain DZ-01-10-aga	100<br>100<br>100	MN220612.1<br>MK955337.1<br>MK577318.1	*Pseudoalteromonas neustonica* PAMC 28425<br>*Pseudoalteromonas prydzensis* MB8-11<br>*Pseudoalteromonas mariniglutinosa* KMM 3635	99.0<br>98.7<br>97.9	NR_151996.1<br>NR_044803.1<br>NR_028992.1	OQ259924
SU136	Inner umbrella	MB	834	*Vibrio splendidus* strain BST398<br>*Vibrio* sp. strain NFH.MB010<br>*Vibrio* sp. hMe27-21	100<br>100<br>100	CP031055.1<br>MG788349.1<br>KX453258.1	*Vibrio lentus* strain CIP 107166<br>*Vibrio lentus* strain 4OM4T<br>*Vibrio kanaloae* strain LMG 20539	100<br>99.9<br>99.8	NR_114982.1<br>NR_028926.1<br>NR_042468.1	OQ259925
SU137	Inner umbrella	MB	757	*Psychrobacter* sp. strain BH36<br>*Psychrobacter* sp. strain GS3<br>*Psychrobacter nivimaris* strain 20_KNBR_Sed_R2	100<br>100<br>100	MN049674.1<br>MN421797.1<br>MN080178.1	*Psychrobacter nivimaris* strain 88/2-7<br>*Psychrobacter adeliensis* strain DSM 15333<br>*Psychrobacter adeliensis* strain SJ 14	100<br>99.7<br>99.7	NR_028948.1<br>NR_117632.1<br>NR_104882.1	OQ259926

SU139	Inner umbrella	HS	768	Alii*Vibrio* sp. strain EL58<br>Alii*Vibrio* sp. H1309/4.1<br>Alii*Vibrio* fischeri, strain: CG2	100<br>100<br>100	MF461376.1<br>LN871555.1<br>AB819695.1	Alii*Vibrio logei*, strain 584<br>*AliiVibrio salmonicida* strain NCIMB 2262<br>Alii*Vibrio logei* strain NCIMB 2252	99.6<br>99.6<br>99.6	LR813682.1<br>NR_116259.1<br>NR_116258.1	OQ259927
SU140	Inner umbrella	HS	756	*Pseudoalteromonas* sp. strain W11<br>*Pseudoalteromonas* sp. strain S49<br>*Pseudoalteromonas nigrifaciens* strain CeD-2	100<br>100<br>100	MN889233.1<br>MN889221.1<br>MN220613.1	*Pseudoalteromonas* arctica A 37-1-2<br>*Pseudoalteromonas* nigrifaciens NBRC 103036<br>*Pseudoalteromonas paragorgicola* KMM 3548	100<br>100<br>100	CP011026.1<br>NR_114188.1<br>NR_025654.1	OQ259928
SU143	Inner umbrella	WSP	1003	*Psychrobacter* sp. P11G5<br>*Psychrobacter glacincola*: NBRC 101053<br>*Psychrobacter alimentarius* strain 1.27	100<br>100<br>99.8	CP012533.1<br>AB681354.1<br>KX417122.1	*Psychrobacter adeliensis* strain DSM 15333<br>*Psychrobacter glacincola* strain DSM 12194<br>*Psychrobacter namhaensis* strain SW-242	98.8<br>98.6<br>98.7	NR_117634.1<br>NR_042076.1<br>NR_043141.1	OQ259929
SU147	Inner umbrella	WSP	835	*Shewanella* sp. HM13<br>*Shewanella livingstonensis* LMG 19866<br>*Shewanella* sp. H2-63	100<br>100<br>100	LC461000.1<br>MK131328.1<br>KM979184.1	*Shewanella livingstonensis* strain LMG 19866<br>*Shewanella vesiculosa* strain M7<br>*Shewanella livingstonensis* strain NF22	100<br>100<br>99.9	CP034015.1<br>NR_042710.1<br>NR_025443.1	OQ259930

Table S2. The IC_{50} values (in µg/mL) of the 16 bacterial strain extracts against ESKAPE pathogens Efm: *Enterococcus faecium*; MRSA, methicillin-resistant *Staphylococcus aureus* and the fish pathogens Lg: *Lactococcus garviae* and Vi: *Vibrio ichthyoenteri*. Positive controls: ampicillin for Efm and Lg, chloramphenicol for MRSA an Vi. n.t: not tested because of the lack of extract. Most potent bioactivities are boldened. Test pathogens and cancer cells which were not susceptible to any of the extracts are not displayed in the table.

Isolate	Media-regime	Activity against Efm	Activity against MRSA	Activity against Lg	Activity against Vi
***Biziona* sp. SU122**	MB-liquid	22.3	20.4	57.6	>100
	MB-solid	60.3	27.7	>100	>100
	GYM-liquid	>100	>100	>100	>100
	GYM-solid	17.3	17.4	82.7	>100
***Psychrobacter* sp. SU123**	MB-liquid	>100	19.6	>100	>100
	MB-solid	>100	21.9	>100	>100
	GYM-liquid	>100	26	>100	>100
	GYM-solid	>100	25.1	>100	>100
***Polaribacter* sp. SU124**	MB-liquid	67.3	**7.3**	>100	>100
	MB-solid	>100	20.9	>100	>100
	GYM-liquid	>100	80.8	>100	>100
	GYM-solid	>100	23.2	>100	>100
***Salinibacterium* sp. SU125**	MB-liquid	>100	>100	>100	>100
	MB-solid	>100	>100	>100	>100
	GYM-liquid	>100	28	>100	56.6
	GYM-solid	>100	66.5	>100	>100
***Shewanella* sp. SU126**	MB-liquid	59.9	20.7	>100	>100
	MB-solid	18.7	**8.5**	53.4	>100
	GYM-liquid	>100	>100	>100	>100
	GYM-solid	n.t	n.t	n.t	>100

***Pseudoalteromonas* sp. SU127**	MB-liquid	42.4	22.4	66.1	>100
	MB-solid	44.7	21.8	72.9	>100
	GYM-liquid	64.6	61.7	>100	>100
	GYM-solid	18.8	18.8	20.7	>100
***Psychrobacter* sp. SU128**	MB-liquid	59.2	21.3	87.4	>100
	MB-solid	79	14.6	>100	>100
	GYM-liquid	19.6	20.4	67.6	>100
	GYM-solid	18.4	12.5	62.7	>100
***Vibrio* sp. SU129**	MB-liquid	62	65	>100	>100
	MB-solid	>100	29.5	>100	91.5
	GYM-liquid	19.7	19.7	72.9	>100
	GYM-solid	20.8	21.3	65	>100
***Polaribacter* sp. SU134**	MB-liquid	>100	>100	>100	>100
	MB-solid	>100	>100	>100	>100
	GYM-liquid	>100	>100	>100	>100
	GYM-solid	n.t	n.t	n.t	>100
***Pseudoalteromonas* sp. SU135**	MB-liquid	>100	62.9	>100	>100
	MB-solid	92.3	26.9	>100	>100
	GYM-liquid	19.2	21.4	63.1	>100
	GYM-solid	18.8	18.3	27.2	>100
***Vibrio* sp. SU136**	MB-liquid	73.7	37.9	>100	>100
	MB-solid	>100	62	>100	>100
	GYM-liquid	>100	>100	>100	>100
	GYM-solid	n.t	n.t	n.t	>100
***Psychrobacter* sp. SU137**	MB-liquid	37.8	21.3	69.6	>100
	MB-solid	19.4	18.7	43.3	>100
	GYM-liquid	18.7	18.5	56.2	>100
	GYM-solid	**7.3**	**8.1**	20.1	>100
***Aliivibrio* sp. SU139**	MB-liquid	>100	63.5	>100	>100
	MB-solid	>100	82.3	>100	>100
	GYM-liquid	>100	>100	>100	>100
	GYM-solid	n.t	n.t	n.t	>100
***Pseudoalteromonas* sp. SU140**	MB-liquid	57.6	20.7	65.3	>100
	MB-solid	58.6	19.4	87.9	>100
	GYM-liquid	17.9	18.1	19.4	>100
	GYM-solid	15.9	17.1	19.5	>100
***Psychrobacter* sp. SU143**	MB-liquid	>100	20.9	>100	>100
	MB-solid	>100	25.7	>100	>100

	GYM-liquid	20.5	20.2	>100	>100
	GYM-solid	39.6	**9.9**	>100	>100
***Shewanella* sp. SU147**	MB-liquid	58.5	42.9	66.9	>100
	MB-solid	19.1	16.2	45.7	>100
	GYM-liquid	17.6	19.4	20.2	>100
	GYM-solid	17.8	18.9	21.9	>100
**Positive control**		1.6	3.1	0.5	0.4

Table S3. Putative annotation of metabolites produced by the SU143, SU137, SU126 and SU124 in the liquid and solid regime of MB and GYM media. Annotation was based on the *m/z* [M+H]+ or other adducts, retention time (t_R), predicted molecular formula, fragmentation pattern and source of the hit. The source of the ion is indicated by the peak area (PkArea) recorded. Confidence level of annotation are given based on the reporting standards (1- 4) proposed by Sumner, *et al.* [1] where 1 is identified compound, 2 is putative annotation without reference standards, 3 is putative characterized compound class, and 4 is unknown compound.

Adduct	parent mass	RTMean	MS²	Mol form.	Hit	Class	PkArea GYM-liquid	PkArea GYM-solid	PkArea MB-liquid	PkArea MB-solid	Confidence/reference
***Psychrobacter* sp. SU143**											
$[M+Na]^+$	511.2096	10.5	414; 391; 367	$C_{30}H_{32}O_6$	-	-	0	0	5386	0	4
$[M+H]^+$	355.4134	8.4	337; 319; 213	$C_{23}H_{46}O_2$	-	Bile acid	0	0	3790	0	3 [2]
$[M+H]^+$	311.2583	7.9	297; 149; 135; 121	$C_{19}H_{34}O_3$	methyl 15-hydroxyoctadeca-9,12-dienoate	fatty acid	0	0	0	7374	3 [3]
$[M+K]^+$	429.2299	8.4	411; 355; 337	$C_{24}H_{39}O_4$	12-hydroxy-3-ketocholanic acid	Bile acid	0	0	7852	0	2 [2]
$[M+Na]^+$	1021.919	10.5	883; 797; 711; 625; 539; 453; 367; 281; 195	$C_{67}H_{120}O_6$	-	PHB	0	0	4681	0	3
$[M+H]^+$	355.4781	8.4	337; 319; 213	-	-	Bile acid	0	0	4991	0	3
$[M+H]^+$	480.3093	9.1	339; 308; 155; 135	$C_{23}H_{47}NO_7P$	PE (18:1/0:0)	lipids	7727	7946	23769	229952	3 [4]
$[M+Na]^+$	849.8456	10.1	797; 711; 625; 539; 453; 367; 281; 195	$C_{55}H_{108}O_5$	-	PHB	0	0	0	3516	3
$[M+H]^+$	220.12	3.9	164; 152; 141	$C_{12}H_{15}N_2O_2$	-		0	0	10835	3915	3
$[M-2H_2O+H]^+$	357.2756	8.3	338; 247; 215; 161	$C_{24}H_{36}O_2$	3,12-dihydroxycholanic acid	Bile acid	0	0	33381	17997	2 [2]
$[M+H]^+$	373.5783	8.4	355; 337; 213	-	-	Bile acid	0	0	1077	0	3
$[M+Na]^+$	942.3873	10.5	625; 539; 453; 367; 295; 281; 195	$C_{48}H_{61}O_{19}$	-	PHB	0	0	0	2229	3

[M+H]+	778.5354	11.1	595; 573; 184; 146	$C_{44}H_{76}NO_8P$	1,2-Di-(9Z,12Z,15Z-octadecatrienoyl)-sn-glycero-3-phosphocholine	lipids	5910	0	0	0	3 [5]
[M+H]+	480.3092	8.9	339; 308; 135; 121	$C_{23}H_{46}NO_7P$	PE (18:1/0:0)	lipids	16667	23682	25275	202226	3 [5]
[M+H]+	339.2897	8.9	301; 265; 247; 149; 135; 121	$C_{21}H_{38}O_3$	methyl 3-hydroxyicosa-11,14-dienoate	fatty acid	6737	10008	10780	127311	3 [3]
[M+H]+	482.3243	9.8	367; 341; 310; 155	$C_{23}H_{49}NO_7P$	PE (18:0/0:0)	lipids	0	0	0	6207	3 [4,5]
[M+H]+	959.6099	9.1	339; 308; 265	-	PE	lipids	0	0	0	9572	3
[M+Na]+	1159.479	8.9	883; 797; 711; 625; 539; 453; 367; 281; 195	$C_{72}H_{70}O_{14}$	-	PHB	0	0	0	33812	3 [6]
[M+H-H2O]+	339.2896	9.1	301; 265; 135; 121;	$C_{21}H_{38}O_3$	Monoelaidin (2,3-dihydroxypropyl (E)-octadec-9-enoate)	fatty acid	948	0	0	65456	3 [7]
[M+Na]+	849.3435	10.1	797; 711; 625; 539; 453; 367; 281; 195	$C_{47}H_{54}O_{13}$	-	PHB	0	0	0	4258	3
[M+H]+	639.5307	10.1	448; 381; 364; 345; 318; 115; 70	$C_{37}H_{70}N_2O_6$	-	ornithine lipids (OL)	7939	0	0	0	3 [8]
[M+H]+	466.2926	8.4	325; 294; 251; 233; 135; 121	$C_{22}H_{44}NO_7P$	lysophosphatidylcholine (14:1/0:0)	lipids	3117	0	2249	20908	3 [5]
[M+H]+	480.4825	9.1	339; 308; 135; 121	-	PE	lipids	0	0	0	3472	3
[M+H]+	452.2774	8.1	311; 280; 237; 219; 135; 121	$C_{21}H_{42}NO_7P$	PE(16:1/0:0)(lysophosphatidylethanolamine	lipids	0	0	0	46551	3 [9]
[M+H]+	268.2642	10.7	251; 233; 149; 135	$C_{17}H_{33}NO$	-	fatty acid	5452	0	0	0	3
[M+H]+	466.2924	8.6	325; 294; 251; 233; 135; 121	$C_{22}H_{44}NO_7P$	lysophosphatidylcholine (14:1/0:0)	lipids	0	0	0	27752	3 [10]
[M+Na]+	643.2577	6.9	557; 539; 471; 453; 385; 367; 299; 281; 213; 195	C28H44O15	27-Hydroxy-3,7,11,15,19,23-hexamethyl-5,9,13,17,21,25-hexaoxo-4,8,12,16,20,24-hexaoxaoctacosanoic acid	PHB	0	0	10183	13347	3 [11]
[M+H]+	373.6283	8.4	355; 213; 199	-	-	Bile acid	0	0	1433	0	3
[M+Na]+	711.284	8.4	625; 539; 453; 367; 281; 195	$C_{32}H_{48}O_{16}$	(4,8,12,16,20,24,28,32)-4,8,12,16,20,24,28,32-Octamethyl-	PHB	0	0	8446	0	3 [11]

					1,5,9,13,17,21,25,29-octaoxacyclodotriacontane-2,6,10,14,18,22,26,30-octone						
$[M+H]^+$	641.5468	10.5	383; 366; 347; 319; 115; 70	$C_{37}H_{72}N_2O_6$	-	ornithine lipids (OL)	21736	0	0	0	3 [5]
$[M+H]^+$	373.4876	8.4				Bile acid	0	0	1531	0	3
$[M+H]^+$	452.2771	7.9	311; 280; 237; 155	$C_{21}H_{42}NO_7P$	PE(16:1/0:0)	lipids	0	0	0	19467	3 [9]
$[M+H]^+$	337.3976	8.4	319; 279; 267; 213; 159	$C_{24}H_{32}O$	-	Bile acid	0	0	998	0	3
$[M+H]^+$	370.2743	6.0	353; 335; 317; 227	$C_{24}H_{35}NO_2$	-	Bile acid	0	0	45902	29963	3
$[M+H-2H_2O]^+$	237.222	11.1	219; 200; 177; 163; 149; 135; 121; 95	$C_{16}H_{32}O_3$	3-Hydroxyhexadecanoic acid	fatty acid	22770	16833	10762	9380	3 [7]
$[M+H]^+$	480.597	9.1	339; 308	-	PE	lipids	0	0	0	1077	3
$[M+H]^+$	522.3555	9.2	184; 124; 104	$C_{26}H_{52}NO_7P$	PC (0:0/18:1)	lipids	1338	0	7505	0	3 [5]
$[M-2H_2O+H]^+$	355.5537	8.4	337; 319; 285; 213	$C_{24}H_{34}O_2$	-	Bile acid	0	0	2779	0	3
	494.324	9.4	353; 339	$C_{24}H_{48}NO_7P$	PE(0:0/19:1)	lipids	0	0	0	2679	3 [5]
$[M+NH_3^+H]^+$	254.2483	10.2	237; 219; 135	$C_{16}H_{28}O$	-	fatty acid	10921	0	0	0	3 [3]
$[M+H]^+$	502.2906	8.9	459; 361; 339; 164; 120	$C_{23}H_{46}NO_7P$	PE (18:1/0:0)	lipids	0	0	1287	17327	3 [5]
$[M+H]^+$	344.2795	9.9	149; 135; 121	$C_{23}H_{36}O_2$	-	fatty acid	4898	0	0	0	3
$[M+H]^+$	372.2896	7.3	355; 337; 319; 213	$C_{21}H_{39}O_5$	-	Bile acid	0	0	30112	18341	3
$[M+Na]^+$	1073.441	8.7	625; 557; 539; 453; 385; 367; 281; 195	$C_{55}H_{70}O_{20}$	-	PHB	0	0	6834	0	3
$[M+H]^+$	406.2953	6.0	370; 353; 335; 317; 253	$C_{24}H_{39}NO_4$	-	Bile acid	0	0	0	14641	3
$[M+H]^+$	482.3243	10.0	341; 310; 285; 109	$C_{23}H_{48}NO_7P$	PE (18:0/0:0)	lipids	0	0	0	8452	3 [5]
$[M+Na]^+$	502.2907	9.1	459; 441; 379; 339; 195; 176; 120	C23H46NO7PNa	1-(9Z-Octadecenoyl)-sn-glycero-3-phosphoethanolamine (PE(18:1/0:0))	lipids	0	0	0	17426	3 [5]
$[M+Na]^+$	942.8887	10.5	639; 625; 539; 453; 367; 281; 195	-	-	PHB	0	0	0	2157	3
$[2M+H]^+$	781.5638	8.4	373; 355; 337; 319	$C_{24}H_{38}O_4$	12-hydroxy-3-ketochalanic acid	Bile acid	0	0	146698	111170	3 [2]
$[M+H]^+$	457.2028	9.0	441; 379; 339; 195; 176	$C_{25}H_{32}NO_5P$	PE	lipids	0	0	0	3304	3

$[M+H]^+$	459.2478	9.1	441; 379; 339; 308; 195; 155	$C_{22}H_{38}NO_7P$	PE	lipids	0	0	0	5061	3
$[M-H2O+H]^+$	373.5322	8.4	355; 337; 319; 213	$C_{24}H_{38}O_4$	12-hydroxy-3-ketocholanic acid	Bile acid	0	0	926	0	3 [2]
$[M+H]^+$	205.0977	2.8	177; 132; 120	$C_{11}H_{12}N_2O_2$	cyclo-(glycinyl-L-phenylalanine)	diketopiperazine	0	0	8194	0	3 [12]
$[M-H]^-$	398:2695	8:4	371; 345; 343; 327; 311	$C_{24}H_{38}O_4$	12-hydroxy-3-ketocholanic acid	Bile acid	0	0	272850	212998	3 [2]
***Psychrobacter* sp. SU137**											
$[2M+H]^+$	781.7834	8.4		$C_{24}H_{38}O_4$	12-Hydrox-3-ketocholanic acid	Bile acid	0	0	3090	0	3 [2]
$[2M+H]^+$	785.5936	8.1	357; 339; 321	$C_{24}H_{40}O_4$	3,12-Dihydroxycholanic acid	Bile acid	0	0	11284	0	3 [2]
$[M+H]^+$	480.3089	9.1	339; 308	$C_{23}H_{46}NO_7P$	PE (18:1/0:0)	lipids	15885	1325	1611	0	3 [5]
$[M-2H_2O+H]^+$	355.4796	8.4	337; 319; 285; 213	-	-	Bile acid	0	0	7991	0	3
$[M+Na]^+$	1101.473	9.7	567; 539; 481; 453; 395; 367; 309; 281; 195	$C_{57}H_{74}O_{20}$	-	PHB	0	61393	0	0	3
$[M+H]^+$	778.5365	10.7	595; 573; 184; 146	$C_{44}H_{76}NO_8P$	1,2-Di-(9Z,12Z,15Z-octadecatrienoyl)-sn-glycero-3-phosphocholine	lipid	1498	0	0	0	3 [5]
$[M+Na]^+$	863.8612	10.8	797; 625; 539; 453; 367; 281; 195	-	-	PHB	0	9031	0	0	3
$[M+Na]^+$	777.8243	10.6	625; 539; 453; 367; 281; 195	-	-	PHB	0	11331	0	0	3
$[M+Na]^+$	820.8437	10.7	711; 625; 539; 453; 367; 309; 281; 195	-	-	PHB	0	18208	0	0	3
$[M-2H_2O+H]^+$	430.2952	7.2	412; 355 337; 319; 213	$C_{26}H_{42}NO_6$	"((4R)-4-((3R,5S,6R,7S,9S,10R,13R,14S,17R)-3,6,7-trihydroxy-10,13-dimethylhexadecahydro-1H-cyclopenta[a]phenanthren-17-yl)pentanoyl)glycine"	Bile acid	0	0	0	89002	3 [7]
$[M-2H_2O+H]^+$	355.2632	10.8	272; 254; 164	$C_{24}H_{36}O_4$	12-hydroxy-3-ketocholanic acid	Bile acid	0	0	1390	0	3 [2]
$[2M+K]^+$	818.108	6.9	355; 337	-	-	Bile acid	0	0	660	0	3

$[M+Na]^+$	1055.431	10.2	969; 883; 797; 711; 625; 539; 453; 367; 281; 195	$C_{55}H_{68}O_{19}$	-	PHB	0	0	5626	0	3
$[M-H_2O+H]^+$	389.269	7.8	353; 325; 307; 147	$C_{24}H_{38}O_5$	dihydroxy-7-ketocholanic acid	Bile acid	0	0	7878	0	3 [2]
$[M+Na]^+$	969.3945	10.0	883; 797; 711; 625; 539; 453; 367; 281; 195	$C_{47}H_{62}O_{20}$	-	PHB	0	0	19654	0	3
$[M+H]^+$	408.3111	8.4	-	$C_{24}H_{41}NO_4$	-	Bile acid	0	0	4615	4740	3
$[M+Na]^+$	453.1734	7.8	367; 281; 195	$C_{20}H_{30}O_{10}$	pentolide (pentaoxacycloeicosanepentone pentamethyl)	PHB	0	42685	0	8166	2 [13]
$[M+Na]^+$	383.1615	4.7	365; 309; 253	$C_{19}H_{24}N_2O_5$	-	-	0	0	18437	0	3
$[M+Na]^+$	501.304	8.8	444; 339; 299	$C_{24}H_{46}O_9$	-	PHB	0	0	0	11713	3
$[M+H]^+$	535.3355	11.1	165	$C_{32}H_{48}O_5$	-	-	0	0	0	7742	3
$[M+H]^+$	367.1371	8.6	227; 213; 145	-	-	Bile acid	0	0	4354	0	3
$[M+H]^+$	337.3968	8.4	319; 267; 213	-	-	Bile acid	0	0	2519	0	3
$[M-2H_2O+H]^+$	337.253	7.1	319; 209; 149	$C_{24}H_{36}O_3$	-	Bile acid	0	0	20058	0	3
$[M+Na]^+$	643.2578	6.9	539; 471; 453; 385; 367; 281; 195	$C_{28}H_{44}O_{15}$	(3,7,11,15,19,23,27)-27-Hydroxy-3,7,11,15,19,23-hexamethyl-5,9,13,17,21,25-hexaoxo-4,8,12,16,20,24-hexaoxaoctacosanoic acid	PHB (linear)	0	0	17603	23999	3 [14]
$[M-2H_2O+H]^+$	357.279	8.4	275; 261; 247; 215	$C_{24}H_{38}O_3$	"(R)-4-((3S,5S,8R,9S,10S,12S,13R,14S,17R)-3,12-dihydroxy-10,13-dimethylhexadecahydro-1H-cyclopenta[a]phenanthren-17-yl)pentanoic acid"	Bile acid	0	0	42563	200354	3 [7]
$[M+Na]^+$	729.295	7.3	643; 625; 539; 471; 453; 367; 281; 195	$C_{32}H_{50}O_{17}$	(3,7,11,15,19,23,27,31)-31-Hydroxy-3,7,11,15,19,23,27-heptamethyl-5,9,13,17,21,25,29-heptaoxo-4,8,12,16,20,24,28-heptaoxadotriacontanoic acid	PHB (linear)	0	15501	17672	0	3 [14]

$[M+H]^+$	426.1863	7.0	355; 337; 319; 213	-	-	Bile acid	0	0	21433	0	3
$[M-2H_2O+H]^+$	371.2584	7.8			"(R)-4-((3S,5S,7R,8R,9S,10S,13R,14S,17R)-3,7-dihydroxy-10,13-dimethyl-12-oxohexadecahydro-1H-cyclopenta[a]phenanthren-17-yl)pentanoic acid"	Bile acid	0	0	11892	0	3
$[M-2H_2O+H]^+$	355.4122	8.4	337; 319; 213	-	-	Bile acid	0	0	10186	0	3
$[M+Na]^+$	843.363	8.9	739; 653; 567; 481; 453; 395; 367; 309; 281; 195	$C_{38}H_{60}O_{19}$	-	PHB	0	11458	0	0	3 [14]
$[M-H_2O+H]^+$	448.3057	7.2	412; 355; 337; 319; 213	$C_{26}H_{43}NO_6$	"((R)-4-((3R,5S,7S,8R,9S,10S,12S,13R,14S,17R)-3,7,12-trihydroxy-10,13-dimethylhexadecahydro-1H-cyclopenta[a]phenanthren-17-yl)pentanoyl)glycine"	Bile acid	0	0	0	49931	3
$[M-H_2O+H]^+$	373.1485	6.9	337; 319; 227; 213; 199	-	-	Bile acid	0	0	1232	845	3
$[M+Na]^+$	470.2878	7.2	452; 395	$C_{26}H_{41}NO_5$	2-(4-(7-hydroxy-9a,11a-dimethyl-4-oxo-hexadecahydro-1H-cyclopenta[a]phenanthren-1-yl)pentanamido)acetic acid	Bile acid	0	0	88851	43472	3
[M-2H2O+H]+	415.2851	8.1	355; 213	$C_{26}H_{42}O_6$	4-[7-(acetyloxy)-4,11-dihydroxy-9a,11a-dimethyl-hexadecahydro-1H-cyclopenta[a]phenanthren-1-yl]pentanoic acid	Bile acid	0	0	0	5490	3
[M+H]+	426.1855	7.2	355; 337; 319; 213	-	-	Bile acid	0	0	6787	0	3

[M+H]+	399.2102	7.5	165	-	-		0	0	0	19139	3
[M+Na]+	987.4049	8.4	797; 711; 625; 539; 453; 367; 299; 281; 195		-	PHB	0	14639	34413	40086	3 [14]
[M+Na]+	711.2843	8.4	625; 539; 453; 367; 281; 195	$C_{32}H_{48}O_{16}$	(4,8,12,16,20,24,28,32)-4,8,12,16,20,24,28,32-Octamethyl-1,5,9,13,17,21,25,29-octaoxacyclodotriacontane-2,6,10,14,18,22,26,30-octone	PHB	0	0	28729	15177	3 [14]
[M+Na]+	625.2478	8.5	539; 453; 367; 281; 195	$C_{28}H_{42}O_{14}$	heptolide	PHB	0	17335	63424	0	3
[M+Na]+	863.3592	10.8	739; 711; 625; 539; 453; 367; 281; 195	-	-	PHB	0	10789	0	0	3
[M+Na]+	883.3577	9.7	797; 711; 625; 539; 453; 367; 281; 195	-	-	PHB	0	13179	15302	0	3
[M+NH4]+	468.3325	8.1	355; 319; 245	$C_{26}H_{42}O_6$	acetylated cholic acid	Bile acid	0	0	8043	0	2 [7]
[M+Na]+	1073.442	8.7	797; 711; 625; 539; 471; 453; 367; 281; 195	-	-	PHB	0	0	11396	0	3
[M-H2O+H]+	373.4902	8.4	355; 337; 319; 213	-	-	Bile acid	0	0	2318	0	3
[M+H]+	480.3088	8.9	-	$C_{23}H_{47}NO_7P$	PE (18:1/0:0)		0	4130	0	0	3
[M+Na]+	557.2209	6.3	453; 385; 367; 299; 281; 213; 195	$C_{24}H_{38}O_{13}$	(3R,7R,11R,15R,19R,23R)-23-Hydroxy-3,7,11,15,19-pentamethyl-5,9,13,17,21-pentaoxo-4,8,12,16,20-pentaoxatetracosanoic acid	PHB (linear)	0	0	58243	13729	3
[2M+Na]+	803.541	8.4				Bile acid	0	0	30723	20893	3
$[M+Na]^+$	797.3203	9.4	711; 625; 539; 453; 367; 281; 195	$C_{36}H_{54}O_{18}$	Cyclic PHB:8	PHB	0	9467	0	0	3 [15]
$[M+Na]^+$	777.3223	10.6	625; 539; 453; 367; 281; 195	$C_{47}H_{58}O_{16}$	-	PHB	0	14488	0	0	3
$[M-2H2O+H]^+$	355.5503	8.4	337; 319; 285; 213	-	-	Bile acid	0	0	4055	0	3
$[M-H2O+H]^+$	373.2753	8.4	355; 337; 319; 213	$C_{24}H_{36}O_3$	12-hydroxy-3-ketocholanic acid	Bile acid	0	0	322629	0	3 [2]

$[M+Na]^+$	901.3686	8.1	815; 625; 539; 453; 367; 299; 281; 195	-	-	PHB	0	14437	41698	0	3
$[M+Na]^+$	1115.488	10.0	539; 453; 367; 281; 195	$C_{72}H_{70}O_{14}$	-	PHB	0	3110	0	0	3 [6]
$[2M+H]^+$	781.5647	8.4	373; 355; 337; 319	$C_{24}H_{38}O_3$	12-hydroxy-3-ketocholanic acid	Bile acid	0	0	186649	116547	3 [2]
$[M+H]^+$	479.3083	12.1	165	$C_{22}H_{38}N_8O_4$	-	-	0	0	0	1205	4
[M-H2O+H]$^+$	387.2896	10.2	369; 355; 337; 319; 213	$C_{25}H_{40}O_4$	Methyl 3-oxo-desoxycholate	Bile acid	0	0	26880	0	3
$[M+Na]^+$	539.2107	8.1	453; 367; 281; 195	$C_{24}H_{36}O_{12}$	Hexolide	PHB	0	0	66179	15227	2 [16]
$[M+Na]^+$	413.2668	8.1	355	$C_{24}H_{38}O_4$	12-hydroxy-3-ketocholanic acid	Bile acid	0	0	0	6445	3 [2]
$[M+Na]^+$	467.2725	9.3	165	$C_{27}H_{40}O_5$	-	-	0	0	0	12988	4
$[M+Na]^+$	470.2882	7.2	452; 395	$C_{26}H_{41}NO_5$	2-(4-(7-hydroxy-9a,11a-dimethyl-4-oxo-hexadecahydro-1H-cyclopenta[a]phenanthren-1-yl)pentanamido)acetic acid	Bile acid	0	0	169519	0	3 [7]
$[M+Na]^+$	929.3994	9.2	739; 653; 567; 481; 453; 367; 309; 281; 195	$C_{53}H_{62}O_{13}$	-	PHB	0	24804	0	0	3
$[M+Na]^+$	820.3415	10.7	711; 625; 539; 453; 367; 281; 195	-	-	PHB	0	22362	0	0	3
$[M+H]^+$	373.4257	8.4	355; 213; 199	-	-	Bile acid	0	0	4825	0	3
$[M+Na]^+$	1187.51	9.9	625; 567; 539; 481; 453; 395; 367; 309; 281; 195	-	-	PHB	0	54908	0	0	3
$[M+H]^+$	411.2462	10.4	165	-	-	-	0	0	0	6038	3
$[M+Na]^+$	711.2845	9.1	625; 539; 453; 367; 281; 195	C32H48O16Na	(4,8,12,16,20,24,28,32)-4,8,12,16,20,24,28,32-Octamethyl-1,5,9,13,17,21,25,29-octaoxacyclodotriacontane-2,6,10,14,18,22,26,30-octone	PHB	0	0	17084	8043	3 [11]
[M-H_2O+H]$^+$	357.2796	8.3	275; 261; 247; 215	$C_{24}H_{38}O_3$	"(R)-4-((5R,8R,9S,10S,13R,14S,17R)-10,13-dimethyl-3-	Bile acid	0	0	0	35811	3 [7]

					oxohexadecahydro-1H-cyclopenta[a]phenanthren-17-yl)pentanoic acid"						
$[M+Na]^+$	815.3313	7.7	711; 625; 539; 453; 367; 299; 281; 195	-	-	PHB	0	15347	6962	0	3
$[M-H]^-$	574.4405	11.2	350; 333	$C_{32}H_{65}NO_5S$	Sulfobacin B	lipids	0	1677	0	0	3 [17]
$[M-H]^-$	398:2695	8:4	371; 345; 343; 327; 311	$C_{24}H_{38}O_4$	12-hydroxy-3-ketocholanic acid	Bile acid	0	0	180236	125290	3 [2]
**SU124**											
$[M+H]^+$	387.3221	6.6	115; 70	$C_{21}H_{42}N_2O_4$	(3-hydroxypentadecanoyl)lysine	OL	6261	0	6705	0	3 [18]
$[M+H]^+$	204.125	3.1	148; 136	$C_{10}H_{13}N_5$	N6-(Delta2-isopentenyl)-adenine	purine	0	22518	0	2524	3 [7]
$[M+H]^+$	336.1671	3.7	204; 148; 136	$C_{15}H_{21}N_5O_4$	N6-Isopentenyladenosine	purine	8217	0	0	0	3 [7]
$[M+H]^+$	312.2536	9.3	219; 135; 121	-	-	Fatty acid	3536	1583	0	0	3
$[M-2H2O+H]^+$	357.2796	8.3	275; 261; 247; 215	$C_{24}H_{38}O_3$	"(R)-4-((3S,5S,8R,9S,10S,12S,13R,14S,17R)-3,12-dihydroxy-10,13-dimethylhexadecahydro-1H-cyclopenta[a]phenanthren-17-yl)pentanoic acid"	Bile acid	0	0	0	91143	3 [7]
$[M-H2O-H]^+$	237.222	10.9	219; 135; 121; 107	$C_{16}H_{30}O_2$	Palmitelaidic acid	Fatty acid	10939	10010	19190	10942	3 [7]
$[M+H]^+$	312.2909	10.4	295; 270; 135	$C_{19}H_{37}NO_2$	Gly-C16:1	Fatty acid	0	0	95841	0	3
$[M+H]^+$	373.306	6.3	115; 70	$C_{20}H_{40}N_2O_4$	(3-hydroxytetradecanoyl)lysine	OL	0	0	28454	12372	3 [19]
$[M+H]^+$	387.322	6.6	115; 70	$C_{21}H_{43}N_2O_4$	(3-hydroxypentadecanoyl)lysine	OL	2689	0	21613	0	3 [19]
$[M+H]^+$	595.5046	10.1	381; 115; 70	$C_{35}H_{66}N_2O_5$	-	OL	13935	10267	108486	0	4 [19]
$[M+H]^+$	211.1453	3.6	183; 138	$C_{11}H_{18}N_2O_2$	cyclo(proline-leucine)	diketopiperazine	0	116989	0	0	2 [12]

[M-H]$^-$	574.4405	11.2	350; 333	$C_{32}H_{65}NO_5S$	Sulfobacin B	lipids	0	0	7165	0	3 [17]

Adduct	parent mass	RTMean	MS^2	Mol form.	Hit	Class	PkArea GYM-liquid	PkArea MB-liquid	PkArea MB-solid	Confidence / Refernce
**SU126**										3
[M+H]$^+$	446.3269	10.8	166; 120	$C_{27}H_{43}NO_4$	Phe-C19:0		0	0	15635	3
M+H-H$_2$O]$^+$	311.2584	10.5	219; 135; 121	$C_{19}H_{36}O_4$	Monopalmitolein		0	984	9450	3 [7]
[M+H-H$_2$O]$^+$	237.2223	10.3	219; 135; 121; 107	$C_{16}H_{30}O_2$	Palmitelaidic acid	Fatty acid	0	0	16237	3 [7]
[M-2H$_2$O+H]$^+$	464.2834	7.6	339; 321; 215; 126	$C_{26}H_{41}NO_4S$	"2-((4R)-4-((3R,5R,9S,10S,12S,13R,14S,17R)-3,12-dihydroxy-10,13-dimethylhexadecahydro-1H-cyclopenta[a]phenanthren-17-yl)pentanamido)ethane-1-sulfonic acid"		0	3759	0	3
[M+H]$^+$	298.2748	9.7	281; 215; 121	$C_{18}H_{35}NO_2$		Fatty acid	0	60396	0	3
[M-2(H$_2$O)+H]$^+$	414.2993	7.2	339; 321; 215	$C_{26}H_{43}NO_5$	glycodeoxycholic acid	Bile acid	0	0	32357	3 [2]
[M+H]$^+$	329.2695	10.3	311; 219; 135	$C_{19}H_{36}O_4$	Monopalmitolein	lipid	0	0	17376	3 [7]
[M+H]$^+$	271.2273	9.2	215; 123; 109	$C_{16}H_{32}O_4$			0	0	24729	3
[M - H$_2$O+H]$^+$	311.2591	10.3	219; 135; 121; 109	$C_{19}H_{36}O_4$	Monopalmitolein	lipid	0	0	26216	3 [7]
[M+H]$^+$	418.2954	9.9	166; 120	$C_{25}H_{39}NO_4$	-	diketopiperazine	0	0	6754	3
[M+H]$^+$	419.3115	5.9	353; 335; 240; 144; 96	$C_{21}H_{42}N_2O_6$	-	-	0	8793	0	3
[M+H]$^+$	254.2485	10.2	237; 219; 149	$C_{16}H_{32}NO$	-	-	0	14315	0	4
[M+H]$^+$	595.5046	10.1	381; 115; 70	C35H66N2O5	-	OL	0	39818	0	4 [19]
[M-H]$^-$	716.5208	9.9	-	$C_{39}H_{75}NO_8P$	PE(16:1/18:0)	lipids	0	2646	0	3 [5]
[M-H]$^-$	574.4405	11.2	350; 333	$C_{32}H_{65}NO_5S$	Sulfobacin B	lipids	0	0	2007	3 [17]

References

1. Sumner, L.W.; Amberg, A.; Barrett, D.; Beale, M.H.; Beger, R.; Daykin, C.A.; Fan, T.W.-M.; Fiehn, O.; Goodacre, R.; Griffin, J.L.; et al. Proposed minimum reporting standards for chemical analysis. *Metabolomics* **2007**, *3*, 211-221, doi:10.1007/s11306-007-0082-2.
2. Li, H.; Shinde, P.B.; Lee, H.J.; Yoo, E.S.; Lee, C.-O.; Hong, J.; Choi, S.H.; Jung, J.H. Bile acid derivatives from a sponge-associated bacterium *Psychrobacter* sp. *Arch. Pharmacal Res.* **2009**, *32*, 857-862, doi:10.1007/s12272-009-1607-1.

3. da Costa, M.S.; Albuquerque, L.; Nobre, M.F.; Wait, R. The identification of fatty acids in bacteria. In *Methods in Microbiology*, Rainey, F., Oren, A., Eds.; Academic Press: 2011; Volume 38, pp. 183-196.
4. Vanounou, S.; Parola, A.H.; Fishov, I. Phosphatidylethanolamine and phosphatidylglycerol are segregated into different domains in bacterial membrane. A study with pyrene-labelled phospholipids. *Mol. Microbiol.* **2003**, *49*, 1067-1079, doi:10.1046/j.1365-2958.2003.03614.x.
5. Sohlenkamp, C.; Geiger, O. Bacterial membrane lipids: diversity in structures and pathways. *FEMS Microbiol. Rev.* **2015**, *40*, 133-159, doi:10.1093/femsre/fuv008.
6. Ma, L.; Zhang, Z.; Li, J.; Yang, X.; Fei, B.; Leung, P.H.; Tao, X. A new antimicrobial agent: poly (3-hydroxybutyric acid) oligomer. *Macromol. Biosci.* **2019**, *19*, 1800432, doi:10.1002/mabi.201800432.
7. Wang, M.; Carver, J.J.; Phelan, V.V.; Sanchez, L.M.; Garg, N.; Peng, Y.; Nguyen, D.D.; Watrous, J.; Kapono, C.A.; Luzzatto-Knaan, T.; et al. Sharing and community curation of mass spectrometry data with Global Natural Products Social Molecular Networking. *Nat. Biotechnol.* **2016**, *34*, 828-837, doi:10.1038/nbt.3597.
8. Kim, S.K.; Park, S.J.; Li, X.H.; Choi, Y.S.; Im, D.S.; Lee, J.H. Bacterial ornithine lipid, a surrogate membrane lipid under phosphate-limiting conditions, plays important roles in bacterial persistence and interaction with host. *Environ. Microbiol.* **2018**, *20*, 3992-4008, doi:10.1111/1462-2920.14430.
9. Czolkoss, S.; Borgert, P.; Poppenga, T.; Hölzl, G.; Aktas, M.; Narberhaus, F. Synthesis of the unusual lipid bis(monoacylglycero)phosphate in environmental bacteria. *Environ. Microbiol.* **2021**, *23*, 6993-7008, doi:10.1111/1462-2920.15777.
10. Vijayakumar, V.; Liebisch, G.; Buer, B.; Xue, L.; Gerlach, N.; Blau, S.; Schmitz, J.; Bucher, M. Integrated multi-omics analysis supports role of lysophosphatidylcholine and related glycerophospholipids in the *Lotus japonicus-Glomus intraradices* mycorrhizal symbiosis. *Plant, Cell Environ.* **2016**, *39*, 393-415, doi:10.1111/pce.12624.
11. Kakasi-Zsurka, S.; Todea, A.; But, A.; Paul, C.; Boeriu, C.G.; Davidescu, C.; Nagy, L.; Kuki, Á.; Kéki, S.; Péter, F. Biocatalytic synthesis of new copolymers from 3-hydroxybutyric acid and a carbohydrate lactone. *J. Mol. Catal. B: Enzym.* **2011**, *71*, 22-28, doi:10.1016/j.molcatb.2011.03.004.
12. de Carvalho, M.P.; Abraham, W.R. Antimicrobial and biofilm inhibiting diketopiperazines. *Curr. Med. Chem.* **2012**, *19*, 3564-3577, doi:10.2174/092986712801323243.
13. Petta, T.; Raichardt, L.; Melo, I.S.; Moraes, L.A.B. Bioassay-guided isolation of a low molecular weight PHB from *Burkholderia* sp. with phytotoxic activity. *Appl. Biochem. Biotechnol.* **2013**, *170*, 1689-1701, doi:10.1007/s12010-013-0292-1.
14. Dalton, B.; Bhagabati, P.; De Micco, J.; Padamati, R.B.; O'Connor, K. A review on biological synthesis of the biodegradable polymers polyhydroxyalkanoates and the development of multiple applications. *Catalysts* **2022**, *12*, 1-44, doi:10.3390/catal12030319.
15. Seebach, D.; Brändli, U.; Müller, H.-M.; Dobler, M.; Egli, M.; Przybylski, M.; Schneider, K. On the macrolactonization of *β*-hydroxy acids. Crystal structures of the pentolide and the hexolide from (*R*)-3-hydroxybutanoic acid. Molecular modeling studies of the tetrolide. *Helv. Chim. Acta* **1989**, *72*, 1704-1717, doi:10.1002/hlca.19890720807.

16. Seebach, D.; Brändli, U.; Schnurrenberger, P.; Przybylski, M. High-yield synthesis of 20-, 24-, and 28-membered macropentolide, -hexolide, and -heptolide, respectively, from (*R*)- or (*S*)-3-hydroxybutanoic acid under Yamaguchi's macrolactonization conditions. *Helv. Chim. Acta* **1988**, *71*, 155-167, doi:10.1002/hlca.19880710119.
17. Maeda, J.; Nishida, M.; Takikawa, H.; Yoshida, H.; Azuma, T.; Yoshida, M.; Mizushina, Y. Inhibitory effects of sulfobacin B on DNA polymerase and inflammation. *Int. J. Mol. Med.* **2010**, *26*, 751-758, doi:10.3892/ijmm_00000522.
18. Kristoffersen, V.; Jenssen, M.; Jawad, H.R.; Isaksson, J.; Hansen, E.H.; Rämä, T.; Hansen, K.Ø.; Andersen, J.H. Two novel lyso-ornithine lipids isolated from an Arctic marine *Lacinutrix* sp. bacterium. *Molecules* **2021**, *26*, 1-14, doi:10.3390/molecules26175295.
19. Elshafie, H.S.; Viggiani, L.; Mostafa, M.S.; El-Hashash, M.A.; Camele, I.; Bufo, S.A. Biological activity and chemical identification of ornithine lipid produced by *Burkholderia gladioli* pv. *agaricicola* ICMP 11096 using LC-MS and NMR analyses. *J. Biol. Res.* **2017**, *90*, 1-8, doi:10.4081/jbr.2017.6534
